# On the control of recurrent neural networks using constant inputs

**Published:** 2025-09-26

**Authors:** Cyprien Tamekue, Ruiqi Chen, ShiNung Ching

**Affiliations:** Department of Electrical and Systems Engineering, Washington University in St. Louis, St. Louis, 63130, MO, USA; Neurosciences Program in the Division of Biology and Biomedical Sciences, Washington University in St. Louis, St. Louis, 63130, MO, USA; Department of Electrical and Systems Engineering, Washington University in St. Louis, St. Louis, 63130, MO, USA

**Keywords:** Control Synthesis, Nonlinear Control, Recurrent Neural Networks, Neurostimulation, Transcranial Direct Current Stimulation

## Abstract

This paper investigates the controllability of a broad class of recurrent neural networks widely used in theoretical neuroscience, including models of large-scale human brain dynamics. Motivated by emerging applications in non-invasive neurostimulation such as transcranial direct current stimulation (tDCS), we study the control synthesis of these networks using constant and piecewise constant inputs. The neural model considered is a continuous-time Hopfield-type system with nonlinear activation functions and arbitrary input matrices representing inter-regional brain interactions. Our main contribution is the formulation and solution of a control synthesis problem for such nonlinear systems using specific solution representations. These representations yield explicit algebraic conditions for synthesizing constant and piecewise constant controls that solve a two-point boundary value problem in state space up to higher-order corrections with respect to the time horizon. In particular, the input is constructed to satisfy a tractable small-time algebraic relation involving the Jacobian of the nonlinear drift, ensuring that the synthesis reduces to verifying conditions on the system matrices. For canonical input matrices that directly actuate k nodes, this implies that the reachable set (with constant inputs) of a given initial state is an affine subspace whose dimension equals the input rank and whose basis can be computed efficiently using a thin QR factorization. Numerical simulations illustrate the theoretical results and demonstrate the effectiveness of the proposed synthesis in guiding the design of brain stimulation protocols for therapeutic and cognitive applications.

## I. Introduction

Non-invasive neurostimulation techniques, such as transcranial magnetic stimulation (TMS) and transcranial electrical stimulation (tES), are increasingly being used to modulate brain activity in order to achieve desired cognitive outcomes [4], [15], [22]. Conceptualizing the brain as a controlled dynamical system [24], [34] offers a powerful framework for identifying key brain regions and networks [26] involved in specific cognitive functions and understanding how they can be modulated using targeted stimulation.

In this regard, there is strong potential at the intersection of network control theory and clinical and basic neuroscience. For instance, there have been successes in the application of linear network control theory to optimize TMS, administered with a constant input over short time intervals [25]. Such applications target the control of specific brain regions based on structural network connectivity derived from diffusion spectrum imaging (DTI). Similarly, at an analysis level, linear network control theory has been employed in human neuroimaging studies, again by leveraging DTI-parameterized models [16]. In this latter study, the authors postulated how specific brain regions may be suitable targets for control based on advantageous controllability properties relative to their potential actuation. A complementary perspective views brain stimulation—particularly deep brain stimulation—as a form of vibrational control that stabilizes desired neural dynamics through high-frequency inputs, a notion that has been formalized and validated in recent theoretical work [27], [28].

While these earlier studies [16], [25] have provided valuable insights by using linear network control theory and structural connectivity data to understand brain dynamics, they are limited in key respects. Foremost, by virtue of linearity, they are only assured to provide local characterizations. In [25], it is shown that these local analyses can offer some predictive power over how stimulation induces functional changes in nonlinear models. However, the changes considered are in terms of low-dimensional correlation metrics between brain areas (network nodes), as opposed to specific configurations in state space. In contrast, our goal is to enable formal control synthesis to induce arbitrary network states in the presence of full nonlinearity, which is likely essential for understanding how different brain dynamics mediate cognitive function.

Indeed, the need for more biologically realistic, nonlinear models of large-scale brain dynamics has been appreciated [5] as a precursor for the application of control theory to cognitive neuroscience. Among the paradigms in this regard are whole-brain Mesoscale Individualized NeuroDynamic (MINDy) models [8], [32]. Our previous works have demonstrated the validity and utility of MINDy models as a generative tool for understanding the relationship between individualized neural architectures and neural dynamics in both resting-state [32], [33] and cognitive task contexts [31]. The MINDy models derived from single-subject resting-state brain imaging data operate at a macroscale level, where each node represents a distinct brain region. A given model captures the temporal evolution of brain activity and the non-linear interactions between hundreds of brain regions, taking the form of a nonlinear dynamical system of continuous-time Hopfield-type recurrent neural networks [18]. All (intrinsic) parameters–the decay and connectivity matrices and the transfer function parameters– are directly estimated from brain activity time series so that the resulting models predict future brain activity, providing a more accurate and biologically plausible representation of large-scale brain dynamics.

A MINDy model belongs to the more general class of Hopfield-type recurrent neural networks, which will constitute the focus of our study herein. Specifically, we consider a model of interconnected neural masses associated with d regions, described by a set of nonlinear differential equations that represent the dynamics of each region’s state over time. The dynamic for the i-th region in this network evolves as

(1) $$\frac{dx_{i}}{dt}(t)=-\alpha_{i}x_{i}(t)+\sum_{j=1}^{d}W_{ij}f_{j}\left(x_{j}(t)\right)+\sum_{j=1}^{k}b_{ij}u_{j}\;\;x_{i}(0)=x_{i}^{0}$$

where $k\in {\mathbb{N}}^{*}$ satisfies k≤d, $x_{i}(t)$ denotes the i-th region’s state at time t≥0, $\alpha_{i}>0$ is a positive parameter reflecting the rate at which the current state of the i-th region decays, $u_{j}$ are constant control inputs, $b_{ij}$ represent coupling coefficients between control inputs and neurons, $W_{ij}$ is a real constant that weights the connection from the j-th region to the i-th region, and $f_{j}$ is the activation function of the j-th region.

**Fig. 1.**
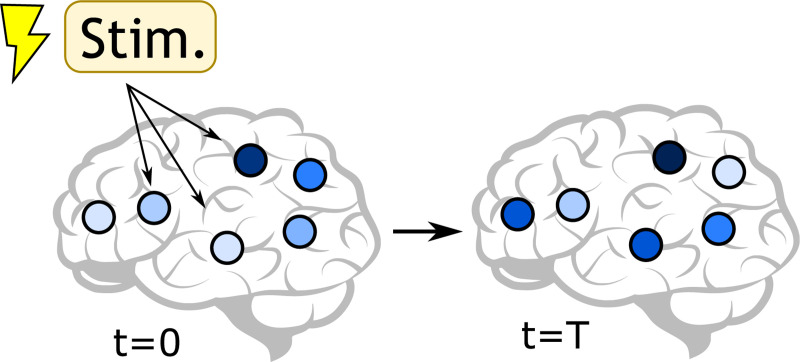
We consider the control of network models of the general form (1). We are motivated by emerging applications in neurostimulation involving the manipulation of brain networks by means of exogenous input.

The study of neural networks, such as (1), has long been a topic of great interest for researchers seeking to understand complex dynamical behaviors and their associated control mechanisms, [3], [13], [18], [36], [40], [41]. Among the various properties investigated, controllability–the ability to steer a network from one state to another within a finite time–stands out for its significant theoretical and practical implications. As highlighted earlier, in fields such as neuroengineering and brain stimulation, particularly transcranial electrical stimulation (tES) with direct current (stimulation via a constant input), the operative issue is to determine a constant input that can drive the state of the network (1) to a desired target state–within the state space–over a short time horizon (Figure 1). Such capability would present a promising avenue for developing stimulation protocols tailored to achieve specific therapeutic outcomes [12], such as altering the excitability of the motor cortex [4].

In previous works such as [13], [36], [40], [41], controllability or stability properties of equations as (1) have been considered in the full-actuated configuration when d=k and $B=\left(b_{ij}\right)=\text{Id}$ or in the specific cases of $\alpha_{i}=0$ for all i, and B belonging to an open dense subset of $M_{d,k}(\mathbb{R})$.

In the current study, we do not impose a priori such a restriction on the input matrix $B\in M_{d,k}(\mathbb{R})$. The assumptions on the activation functions are relaxed to those commonly used in the literature in studying neural networks. The general consideration on B and the complexity arising from the inherent nonlinear dynamics present significant challenges, and systematic solutions for the *control synthesis* problem are not readily available in the current literature. To address this challenge, we derive an explicit algebraic representation for the hidden state x(t) and the associated constant input that solves the two-point boundary value problem in state space up to higher-order terms. This yields an explicit small-time algebraic condition for the constant input, showing that the required control can be determined by inverting an expression involving the drift’s Jacobian and the desired change in state, up to higher-order corrections with respect to the time horizon. From this derivation, we then provide a step function (piece-wise constant control) for large periods of time. Although tDCS typically relies on constant or slowly modulated inputs, step constant controls provide a theoretically grounded and numerically efficient extension of constant-input synthesis over longer time horizons. Note that, in the case of linear activation functions, the synthesized constant control remains valid for both short and long time horizons.

For input matrices that directly actuate k nodes, we show that the reachable set with constant inputs is an affine subspace whose dimension equals the rank of B, and its basis can be computed explicitly via a thin QR factorization of the associated constraint matrix. This characterization applies to both linear and nonlinear activation functions, clarifying how the local structure of the input matrix shapes the short-horizon reachable set.

The remainder of the paper is organized as follows. In the next section, we introduce the general notations used throughout the paper. Section II presents our modeling framework, reformulating equation (1) into a form more suitable for controllability analysis and establishing key results concerning solution representations. Section III addresses the control synthesis problem and is divided into several parts: Section III-A focuses on the linear activation case; Section III-B develops control synthesis strategies for general nonlinear activations that appear to be the proper generalization of the linear case; and Section III-C outlines a taxonomy of synthesis approaches derived from dual representations of the solution, clarifying the conceptual origin of the forward nominal-state and backward nominal-state strategies. Section III-D then provides practical considerations by analyzing the structure of reachable sets associated with the proposed step-function control syntheses. Section IV offers broader discussion and possible extensions. Section V presents numerical illustrations of the theory. Finally, Section VI summarizes the main results and discusses directions for future research. Technical proofs of some results are deferred to the Appendix.

### Notation 1.

In this paper, ℤ stands to the set of integers, ${\mathbb{N}}^{*}$ denote the set of positive integers and $\mathbb{N}={\mathbb{N}}^{*}\cup \left\{0\right\}$. For all a, b∈ℕ, we denote by [​[a,b]​]=ℕ∩[a,b]. For all d, $k\in {\mathbb{N}}^{*}$, we denote by $M_{d,k}(\mathbb{R})$ the set of d×k matrices with real coefficients. ${\mathbb{R}}^{d}$ denotes the d-dimensional real column vector and $L\left({\mathbb{R}}^{d}\right)$ the space of linear maps on ${\mathbb{R}}^{d}$ that we identify, in the usual way, with $M_{d}(\mathbb{R})$ the set of squared matrices of order d with real coefficients. For all $(x,y)\in {\left({\mathbb{R}}^{d}\right)}^{2}$, we denote by |x| the Euclidean norm of x and by 〈x,y〉 the scalar product of x and y. We denote the identity matrix in $M_{d}(\mathbb{R})$ by Id, and for every matrix $M\in M_{d,k}(\mathbb{R})$, $\|M\|=\sup\left\{\left|Mx\;|\;|\;x\in {\mathbb{R}}^{k},\;|x|=1\right\}\right.$ denotes the spectral norm of M, and $M^{†}$ denotes the Moore-Penrose pseudo-inverse of M. For a symmetric matrix $M\in M_{d}(\mathbb{R})$, $\lambda_{\min}(M)$ and $\lambda_{\max}(M)$ denote respectively its smaller and largest eigenvalues. For ease of notation, a diagonal matrix $\text{Γ}=\mathrm{diag}\left(\gamma_{1},\gamma_{2},\cdots ,\gamma_{d}\right)\in M_{d}(\mathbb{R})$, will simply denoted as $\text{Γ}=\left(\gamma_{i}\delta_{i,j}\right)$, where $\delta_{i,j}$ is the usual Kronecker symbol. If $A\in M_{d}(\mathbb{R})$, then $e^{A}\in M_{d}(\mathbb{R})$ and σ(A) are, respectively, the exponential and the set of eigenvalues of A, viz.


(2) $$e^{A}=\sum_{n=0}^{\infty }\frac{A^{n}}{n!},\;\sigma (A)=\{\lambda \in \mathbb{C}|\ker(A-\lambda \text{Id})\neq 0\}.$$

We recall that O stands to the big O-notation, and $y(t)\underset{t\sim 0}{=}O(x(t))$ means y(t) is of the order of x(t). Finally, we use $\dot{x}(t)=\frac{dx}{dt}(t)$ for the total derivative of x w.r.t. t.

## II. Representation of solutions

To study the controllability of (1), it is convenient to recast it in abstract form. Introduce the (nonlinear) map

(3) $$N(x)=-Dx+Wf(x)\;\forall x\in {\mathbb{R}}^{d}.$$

Then, (1) recasts as

(4) $$\dot{x}(t)=N(x(t))+Bu,\;x(0)=x^{0}$$

where $x={\left(x_{1},x_{2},\cdots ,x_{d}\right)}^{⊤}\in {\mathbb{R}}^{d}$ is the state’s vector, $x^{0}={\left(x_{1}^{0},\cdots ,x_{d}^{0}\right)}^{⊤}\in {\mathbb{R}}^{d}$ is the initial state, $u={\left(u_{1},\cdots ,u_{k}\right)}^{⊤}\in {\mathbb{R}}^{k}$ is a constant control, $B=\left(b_{ij}\right)\in M_{d,k}(\mathbb{R})$ is the input matrix, $D=\left(\alpha_{i}\delta_{i,j}\right)\in M_{d}(\mathbb{R})$ is the decay matrix, $W=\left(W_{ij}\right)\in M_{d}(\mathbb{R})$ is the connectivity matrix and $f:{\mathbb{R}}^{d}\to {\mathbb{R}}^{d}$ given by $f(x)={\left(f_{1}\left(x_{1}\right),f_{2}\left(x_{2}\right),\cdots ,f_{d}\left(x_{d}\right)\right)}^{⊤}$ is the firing rate function of the network.

### Notation 2.

We set A=−D+W, where D is the decay matrix and W is the connectivity matrix in (3).

### Assumptions II.1.

Unless otherwise stated, we assume that for every i∈[​[1,d]​], the activation function $f_{i}:\mathbb{R}\to \mathbb{R}$ is a $C^{2}$, globally Lipschitz function on ℝ with a bounded second derivative, and satisfies $f_{i}(0)=0$. The latter is without loss of generality since we may always take for every s∈ℝ, ${\tilde{f}}_{i}(s)=f_{i}(s)-f_{i}(0)$ and set $\tilde{Bu}=Bu+Wf(0)$ as the new input to equation (4). Finally, for the sake of simplicity in the presentation, we also assume that ${\left\|f_{i}^{\prime }\right\|}_{\infty }=1$. Indeed, as long as ${\left\|f_{i}^{\prime }\right\|}_{\infty }\neq 0$, we can always define an activation function ${\tilde{f}}_{i}(s)=f_{i}(\lambda s)$ with $\lambda =1/{\left\|f_{i}^{\prime }\right\|}_{\infty }$ and s∈ℝ.

Throughout the following, we will refer to the following fixed parameters:

$$\begin{array}{l} \text{Γ}=\lambda_{\min}(D)-\|W\|,\;\text{Λ}=\lambda_{\max}(D)+\|W\|,\\ {\text{Λ}}_{1}=\|W\|{\left\|f^{\prime \prime }\right\|}_{\infty }\;\text{and}\;{\left\|f^{\prime \prime }\right\|}_{\infty }=\underset{i\in [\text{​}[1,d]\text{​}]}{\max}{\left\|f_{i}^{\prime \prime }\right\|}_{\infty }. \end{array}$$

Recall that the vector field N is globally Lipschitz on ${\mathbb{R}}^{d}$ (see, for instance, Lemma A.1). Denoting by $\phi_{t}:{\mathbb{R}}^{d}\to {\mathbb{R}}^{d}$ its flow at t∈ℝ, it follows that the family $\left\{\phi_{t}|t\in \mathbb{R}\right\}$ is a one parametric subgroup of $\text{Diff}\left({\mathbb{R}}^{d}\right)$, the group of diffeomorphism of ${\mathbb{R}}^{d}$. Note that for any $x^{0}\in {\mathbb{R}}^{d}$, $\phi_{t}\left(x^{0}\right)$ is the unique solution of (4) when the control u=0, namely

(5) $$\frac{d}{dt}\phi_{t}\left(x^{0}\right)=N\left(\phi_{t}\left(x^{0}\right)\right),\;\phi_{0}\left(x^{0}\right)=x^{0}.$$

Let $\psi_{t}$ denote the inverse of $\phi_{t}$, i.e., $\psi_{t}={\left(\phi_{t}\right)}^{-1}=\phi_{-t}$. Then, for every $x^{0}\in {\mathbb{R}}^{d}$, it holds

$$\frac{d}{dt}\psi_{t}\left(x^{0}\right)=-N\left(\psi_{t}\left(x^{0}\right)\right),\;\psi_{0}\left(x^{0}\right)=x^{0}.$$

In particular, the map $(t,x)\in \mathbb{R}\times {\mathbb{R}}^{d}\mapsto \phi_{t}(x)\in {\mathbb{R}}^{d}$ is of class $C^{2}$ (see, for instance, [17, Chapter 15, Theorem 1]). Moreover, for a fixed t∈ℝ, if we use $D\phi_{t}(x)$ to denote the differential of $\phi_{t}$, then $D\phi_{t}(x)$ is a well-defined invertible matrix, and it holds

(6) $${\left[D\phi_{t}(x)\right]}^{-1}=D\psi_{t}\left(\phi_{t}(x)\right),\;\forall x\in {\mathbb{R}}^{d}.$$

The classical theory of ordinary differential equations (ODE) can be invoked to justify the existence and uniqueness of solutions of (4) (see, for instance, [6, Chapter 2]). In this work, we represent the system’s state trajectory in a form reminiscent of that of linear time-invariant systems as used in [38, Theorem 4.4]. This representation emerged from the chronological calculus framework introduced by Agrachev and Gamkrelidze in the late 1970s for solving nonautonomous ODEs on finite-dimensional manifolds [2]. For a comprehensive treatment of this theory, see also [1, Chapter 6], and [21], [30] for applications in geometric control theory. This representation is noteworthy because, in the specific case of linear activation functions, it naturally aligns with the variation of the constants formula. From this perspective, the proposed representation and approach are a proper generalization of the variation of constants formula to the case of models of the form (4).

The first result of this paper is about solution representation, and it states as follows. The proof is presented in Section B-A.

### Theorem II.2.

*Let* T>0 *and* $\left(x^{0},u\right)\in {\mathbb{R}}^{d}\times {\mathbb{R}}^{k}$. *The solution* $x\in C^{3}\left([0,T];{\mathbb{R}}^{d}\right)$ *of* (4) *can be expressed as*

(7) $$x(t)=\phi_{t}\left(x^{0}+\int_{0}^{t}D\psi_{s}(x(s))Buds\right),\;\forall t\in [0,T].$$

When the dynamics are linear, this representation reduces to the familiar form.

### Corollary II.3.

*Let* T>0 *and* $\left(x^{0},u\right)\in {\mathbb{R}}^{d}\times {\mathbb{R}}^{k}$. *If* $f_{i}(s)=s$, *the solution* $x\in C^{\infty }\left([0,T];{\mathbb{R}}^{d}\right)$ *to* (4) *reads*

(8) $$x(t)=e^{tA}x^{0}+\int_{0}^{t}e^{(t-s)A}Buds\;\forall t\in [0,T].$$

*Proof.* In this case, N(x)=Ax for every $x\in {\mathbb{R}}^{d}$ where A is introduced in Notation 2. In this case, $\phi_{t}(x)=e^{tA}x$ and $\psi_{t}(x)=e^{-tA}x$ for every t∈ℝ. It follows that $D\psi_{t}(x(t))=e^{-tA}$ and the result then follows by (7). □

We conclude this section by presenting a second representation of the solution to (4). It can be viewed as a natural extension of the linear case given in Corollary II.3, particularly at the final time horizon T. The proof follows similar lines to that of Theorem II.2.

### Theorem II.4.

*Let* T>0 *and* $\left(x^{0},u\right)\in {\mathbb{R}}^{d}\times {\mathbb{R}}^{k}$. *Then the solution* $x\in C^{3}\left([0,T];{\mathbb{R}}^{d}\right)$ *of* (4) *can be expressed as*

(9) $$x(t)=\psi_{T-t}\left(\phi_{T}\left(x^{0}\right)+\int_{0}^{t}D\phi_{T-s}(x(s))Buds\right)$$

*for all* t∈[0,T].

### Remark II.5.

Although Theorems II.4 and II.2 are stated for constant inputs $u\in {\mathbb{R}}^{k}$, they remain valid for time-dependent inputs $u\in L^{1}\left([0,T];{\mathbb{R}}^{k}\right)$. Note that this is a specific case of [37, Theorem 3.2]. In this case, the corresponding solution x(⋅) to (4) belongs to $C^{0}\left([0,T];{\mathbb{R}}^{d}\right)$. This observation is particularly relevant, as we will later consider step constant controls—i.e., piecewise constant controls defined on [0,T].

## III. Controllability of the neural networks with step function

In this section, we analyze the controllability of (4) under constant inputs over short time horizons and extend the approach to step-function controls for longer-horizon transfers in the fully nonlinear setting. When the activation functions are linear, the synthesized constant control remains valid for both short and long time horizons.

Recall, e.g., from [39, Remark 4.2.3] that a step function on [0,T] is a piecewise constant function defined over [0,T], i.e., constant on each interval of a partition of [0,T]. Then, a constant function corresponds to a special case of a step function with a single constant value across the entire interval.

Let us introduce the following.

### Definition 3 (Step controllability).

Let T>0. System (4) is step controllable over the time interval [0,T] if, for all $x^{0}$, $x^{1}\in {\mathbb{R}}^{d}$, there exists a step function u on [0,T] such that the solution of (4) with $x(0)=x^{0}$ satisfies $x(T)=x^{1}$.

#### A. The case of linear activation functions

To gain insight into the controllability of the nonlinear system (4) under constant control, it is instructive to first analyze the corresponding linearized model—a strategy that has been clearly articulated and effectively motivated in prior work, notably by [19].

Consider first the linear neural network

(10) $$\dot{x}(t)=Ax(t)+Bu,\;x(0)=x^{0}\in {\mathbb{R}}^{d},$$

with A=−D+W (corresponding to $f_{i}(s)=s$ for all i) and constant input $u\in {\mathbb{R}}^{k}$. System (10) is controllable on [0,T] if and only if the Kalman rank condition holds. Equivalently, the invertibility of the controllability Gramian also guarantees controllability over [0,T]. Moreover, when these conditions are met, one can synthesize a time-varying control $\underline{u}\in L^{\infty }\left((0,T);{\mathbb{R}}^{k}\right)$ of minimal $L^{2}$-energy that steers (10) from any $x^{0}$ to any $x^{1}$ in time T (see, e.g., [10], [39]).

However, this synthesis does not systematically extend to *constant control* capable of achieving the same objective. If the system is controllable, and we assume the existence of a constant input $\tilde{u}\in {\mathbb{R}}^{k}$ that steers the system from $x^{0}\in {\mathbb{R}}^{d}$ to $x^{1}\in {\mathbb{R}}^{d}$ over the interval [0,T], then from (8) and $x(T)=x^{1}$,

$$\int_{0}^{T}e^{(T-s)A}dsB\tilde{u}=x^{1}-e^{TA}x^{0}$$

so that left multiplying by A and using the identity

(11) $$A\int_{0}^{T}e^{(T-s)A}ds=\int_{0}^{T}\left(-\frac{d}{ds}e^{(T-s)A}\right)ds=e^{TA}-\text{Id}$$

we find

(12) $$\left(e^{TA}-\text{Id}\right)B\tilde{u}=A\left(x^{1}-e^{TA}x^{0}\right).$$

However, (12) can be ill-posed, as shown by the following counter-example.

### Remark III.1.

Consider the case where k=d=2, T=2π/ω, B=Id, and

$$A=\left[\begin{array}{cc} 0 & -\omega \\ \omega & 0 \end{array}\right],\;e^{TA}=\left[\begin{array}{cc} \cos(2\pi ) & -\sin(2\pi )\\ \sin(2\pi ) & \cos(2\pi ) \end{array}\right]=\text{Id}.$$

The linear system (10), equipped with time-varying control, is fully actuated and hence controllable, as guaranteed by the Kalman rank condition. However, any constant control $\tilde{u}\in {\mathbb{R}}^{2}$ must satisfy (12). Since $e^{TA}-\text{Id}=0$, it follows that no constant control $\tilde{u}\in {\mathbb{R}}^{2}$ can steer $x^{0}=0$ to a nonzero target state $x^{1}\neq 0$ over the interval [0,T]. Notably, T=2π/ω can be made arbitrarily small by choosing ω≫2π.

On the other hand, using the Gramian-based synthesis, one finds that $W_{c}^{-1}=\text{Id}/T$, and

$$\underline{u}(t)=\frac{1}{T}e^{(T-t)A^{⊤}}x^{1},\;t\in [0,T]$$

is the time-varying control of minimal $L^{2}$-norm that steers $x^{0}=0$ to $x^{1}\neq 0$. In particular, $\|\underline{u}{\|}_{L^{2}}^{2}=\langle W_{c}^{-1}x^{1},x^{1}\rangle ={\left|x^{1}\right|}^{2}/T$. Taking $x^{1}={\left(\sqrt{T/\omega },0\right)}^{⊤}$ yields $\|\underline{u}{\|}_{L^{2}}^{2}=1/\omega$, showing that if ω≫1, then $\underline{u}$ achieves the transfer from $x^{0}=0$ to $x^{1}={\left(\sqrt{T/\omega },0\right)}^{⊤}$ in T>0 with small effort.

Remark III.1 suggests that the controllability of a linear system does not imply the existence of a constant control that solves the control objective.

### Proposition III.2.

*Let* T>0 *and* $\left(x^{0},x^{1}\right)\in {\left({\mathbb{R}}^{d}\right)}^{2}$. *Assume that for any* ℓ∈ℤ, $i\frac{2\pi \ell }{T}\notin \sigma (A)$. *Then, the solution* x(⋅) *to* (10) *satisfies* $x(T)=x^{1}$, *if and only if*, $u\in {\mathbb{R}}^{k}$ *solves*

(13) $$Bu={\left(e^{TA}-\text{Id}\right)}^{-1}A\left(x^{1}-e^{TA}x^{0}\right).$$

The proof of the following result is immediate.

### Corollary III.3.

*Under hypotheses of Proposition III.2*, (13) *has at least one solution* $u\in {\mathbb{R}}^{k}$, *if and only if*,

$${\left(e^{TA}-\text{Id}\right)}^{-1}A\left(x^{1}-e^{TA}x^{0}\right)\in \text{Im}\;B.$$

*In this case*, $u\in {\mathbb{R}}^{k}$ *given by*

$$u=B^{†}{\left(e^{TA}-\text{Id}\right)}^{-1}A\left(x^{1}-e^{TA}x^{0}\right)$$

*is the corresponding least-norm constant control*.

*Proof of Proposition III.2*. Under the assumption on the eigenvalues of A, the matrices A and $e^{TA}-\text{Id}$ are invertible. By Corollary II.3, the solution $x\in C^{\infty }\left([0,T];{\mathbb{R}}^{d}\right)$ of (10) with a constant control $u\in {\mathbb{R}}^{k}$ is given by (8). Let us show that $x(T)=x^{1}$ is equivalent to (13). If $x(T)=x^{1}$, then one obtains immediately that $u\in {\mathbb{R}}^{k}$ solves (13) by invoking the same arguments used to derive (12). Conversely, if (13) is satisfied, one deduces from (11) and (8) that

$$Ax(T)=Ae^{TA}x^{0}+\left(e^{TA}-\text{Id}\right)Bu=Ax^{1}$$

so that $x(T)=x^{1}$ since A is invertible. □

### Remark III.4.

If $\left\|W\|<\lambda_{\min}(D\right)$, then the matrix $e^{TA}-\text{Id}$ is invertible for all T>0, as ensured by Lemma C.2 and the Neumann series expansion. Moreover, for all $x\in {\mathbb{R}}^{d}$, we have

$$\left|Ax|\geq |Dx|-|Wx|\geq \left(\lambda_{\min}(D)-\|W\|\right)|x\right|$$

which implies that A is invertible as well.

Therefore, in Proposition III.2, the spectral assumptions become relevant only when $\left\|W\|\geq \lambda_{\min}(D\right)$.

### Remark III.5.

Remark III.1 and Proposition III.2 highlight a key distinction between constant and time-varying control synthesis in linear systems.

While time-varying control synthesis depends solely on the structural properties of the pair (A,B)–as captured by the invertibility of the controllability Gramian—constant control synthesis is more restrictive. In particular, it depends not only on the matrices A and B, but also crucially on the time horizon T>0, the initial and target states $x^{0}$ and $x^{1}$, and on the spectrum of A. As shown in Remark III.1, even a fully actuated system may fail to connect certain states under constant input when $e^{TA}=\text{Id}$, rendering (12), then (13) unsolvable.

#### B. The case of nonlinear activation functions

Proposition III.2 highlights that controllability under constant control depends intricately on the time horizon T, the spectrum of DN(⋅), and the initial and target states $x^{0}$ and $x^{1}$.

Based on the solution representations of (4) given in Theorems II.2 and II.4, we note that the latter naturally extends the linear case, particularly at the final time horizon T. Nonetheless, both formulations provide valid foundations for synthesizing controls that achieve the desired state transfer. The key distinction lies in their temporal structure and directionality, as well as in the specific conditions required to guarantee their applicability.

##### 1) Forward nominal-state synthesis:

Building on the solution representation of (4) given in Theorem II.4, we aim to synthesize step-function controls that achieve the control objective. Unlike the linear case, the control is first derived in an implicit form due to the nonlinear nature of the system. Then, under the assumption of a small time horizon, we provide an explicit expression that is an accurate approximation of the step-function controls that solve the control objective.

We begin by stating a key result on which the constant control synthesis is based. The proof is identical to that presented in Section C-A.

### Proposition III.6.

*Let* T>0 *and* $\left(x_{0},u\right)\in {\mathbb{R}}^{d}\times {\mathbb{R}}^{k}$. *The solution* x(⋅) *of* (4) *can be expanded at time* t=T *as*

(14) $$\begin{array}{l} x(T)=\sum_{n=0}^{\infty }{\left(-1\right)}^{n}\frac{T^{n+1}DN{\left(\phi_{T}\left(x^{0}\right)\right)}^{n}}{(n+1)!}D\phi_{T}\left(x^{0}\right)Bu\\ \;+\phi_{T}\left(x^{0}\right)-\varphi_{u}(T)Bu. \end{array}$$

*Here* $\varphi_{u}(T)=\kappa_{u}(T)+\eta_{u}(T)$,

(15) $$\kappa_{u}(T)=\sum_{n=1}^{\infty }\int_{0}^{T}\frac{{\left(t-T\right)}^{n+1}}{(n+1)!}\left[\frac{d}{dt}Z_{T-t}^{n}\right]Q_{T-t}dt,$$

(16) $$\eta_{u}(T)=\sum_{n=1}^{\infty }\int_{0}^{T}\frac{{\left(t-T\right)}^{n}Z_{T-t}^{n-1}}{n!}D^{2}\phi_{T-t}(x(t))\dot{x}(t)dt$$

*where* $Q_{T-t}=D\phi_{T-t}(x(t))$, $Z_{T-t}=DN\left(\phi_{T-t}(x(t))\right)$ *and* $D^{2}\phi_{T-t}(x(t))$ *is the second derivative of* $\phi_{T-t}$ *at* x(t).

The implicit constant control synthesis of this section is therefore presented in the following theorem.

### Theorem III.7.

*Let* T>0 *and* $\left(x^{0},x^{1}\right)\in {\left({\mathbb{R}}^{d}\right)}^{2}$. *Assume that for any* ℓ∈ℤ, $i\frac{2\pi \ell }{T}\notin \sigma \left(DN\left(\phi_{T}\left(x^{0}\right)\right)\right)$. *Then, the solution* x(⋅) *to* (4) *satisfies* $x(T)=x^{1}$, *if and only if*, $u\in {\mathbb{R}}^{k}$ *solves*

(17) $$\left[\mathrm{Id}-A_{T}\left(x^{0}\right)\varphi_{u}(T)\right]Bu=A_{T}\left(x^{0}\right)\left(x^{1}-\phi_{T}\left(x^{0}\right)\right).$$

*Here* $\varphi_{u}(T)\in M_{d}(\mathbb{R})$ *is defined in Proposition III.6, and letting* $U_{T}\left(x^{0}\right)=DN\left(\phi_{T}\left(x^{0}\right)\right)$, *one has*

(18) $$A_{T}\left(x^{0}\right)=D\psi_{T}\left(\phi_{T}\left(x^{0}\right)\right){\left(\mathrm{Id}-e^{-TU_{T}\left(x^{0}\right)}\right)}^{-1}U_{T}\left(x^{0}\right).$$

*Proof.* Let us show that $x(T)=x^{1}$ is equivalent to (17). If $x(T)=x^{1}$, then left multiplying (14) by $U_{T}\left(x^{0}\right)$, using (2), and

$$\sum_{n=0}^{\infty }\frac{{\left(-TU_{T}\left(x^{0}\right)\right)}^{n+1}}{(n+1)!}=\sum_{n=1}^{\infty }\frac{{\left(-TU_{T}\left(x^{0}\right)\right)}^{n}}{n!}=e^{-TU_{T}\left(x^{0}\right)}-\text{Id}$$

yields

(19) $$\begin{array}{l} U_{T}\left(x^{0}\right)\left(x^{1}-\phi_{T}\left(x^{0}\right)\right)=-U_{T}\left(x^{0}\right)\varphi_{u}(T)Bu\\ \;+\left(\mathrm{Id}-e^{-TU_{T}\left(x^{0}\right)}\right)D\phi_{T}\left(x^{0}\right)Bu. \end{array}$$

Since ${\left[D\phi_{T}\left(x^{0}\right)\right]}^{-1}=D\psi_{T}\left(\phi_{T}\left(x^{0}\right)\right)$, we left multiply (19) respectively by ${\left(\text{Id}-e^{-TU_{T}\left(x^{0}\right)}\right)}^{-1}$ and $D\psi_{T}\left(\phi_{T}\left(x^{0}\right)\right)$ to get

$$A_{T}\left(x^{0}\right)\left(x^{1}-\phi_{T}\left(x^{0}\right)\right)=\left[\mathrm{Id}-A_{T}\left(x^{0}\right)\zeta_{u}(T)\right]Bu$$

which is exactly (17) where $A_{T}\left(x^{0}\right)$ is defined by (18). Conversely, assume that $u\in {\mathbb{R}}^{k}$ solves (17). Then, one finds

(20) $$A_{T}\left(x^{0}\right)\varphi_{u}(T)Bu=Bu-A_{T}\left(x^{0}\right)\left(x^{1}-\phi_{T}\left(x^{0}\right)\right).$$

One the other hand, from (14), one deduces that

(21) $$A_{T}\left(x^{0}\right)\varphi_{u}(T)Bu=Bu-A_{T}\left(x^{0}\right)\left(x(T)-\phi_{T}\left(x^{0}\right)\right).$$

Identifying (20) with (21), one gets

(22) $$A_{T}\left(x^{0}\right)\left(x(T)-x^{1}\right)=0.$$

Since $A_{T}\left(x^{0}\right)$ is invertible, it follows from (22) that $x(T)=x^{1}$. This completes the proof of the necessary part. □

The synthesis provided in Theorem III.7 is implicit, as the operator $\varphi_{u}(T)$ depends on the state trajectory x(⋅), and thus on the control input u. This dependence poses practical limitations, even when a solution exists. In applications such as brain stimulation via tDCS, one is often interested in modulating activity over short time horizons. In what follows, we derive an expansion of the matrix $\varphi_{u}(T)$ in the small-time regime that enables accurate synthesis in small time horizons. The proof of the following is presented in Section C-B.

### Proposition III.8.

*Under the assumptions of Theorem III.7, the following expansion holds*

(23) $$\varphi_{u}(T)\underset{T\sim 0}{=}\left(\mathrm{Id}-e^{-TU_{T}\left(x^{0}\right)}\right)U_{T}{\left(x^{0}\right)}^{-1}D\phi_{T}\left(x^{0}\right)\;\;-\left(e^{TU_{T}\left(x^{0}\right)}-\text{Id}\right)U_{T}{\left(x^{0}\right)}^{-1}+O\left(T^{3}\right)$$

*In particular, the following expansion also holds*

(24) $$\mathrm{Id}-A_{T}\left(x^{0}\right)\varphi_{u}(T)\underset{T\sim 0}{=}D\phi_{T}{\left(x^{0}\right)}^{-1}e^{-TU_{T}\left(x^{0}\right)}+O\left(T^{2}\right).$$

### Remark III.9.

It is worth noting that the $O\left(T^{3}\right)$ term in (23) becomes $O\left(T^{2}\right)$ in (24) due to the relation

$$A_{T}\left(x^{0}\right)O\left(T^{3}\right)\underset{T\sim 0}{=}O\left(T^{2}\right),$$

since $A_{T}\left(x^{0}\right)$ includes the factor ${\left(\text{Id}-e^{-TU_{T}\left(x^{0}\right)}\right)}^{-1}$, which behaves like $O\left(T^{-1}\right)$ as T→0, given that $U_{T}\left(x^{0}\right)$ is uniformly bounded with respect to T>0 and $x^{0}$.

The explicit constant control synthesis of this section is summarized in the following theorem, which includes an estimate of the endpoint error.

### Theorem III.10.

*Let* T>0 *and* $\left(x^{0},x^{1}\right)\in {\left({\mathbb{R}}^{d}\right)}^{2}$. *Assume that for any* ℓ∈ℤ, $i\frac{2\pi \ell }{T}\notin \sigma \left(DN\left(\phi_{T}\left(x^{0}\right)\right)\right)$. *Then, the solution* x(⋅) *to* (4) *satisfies* $x(T)=x^{1}$, *if and only if*, $u\in {\mathbb{R}}^{k}$ *solves*

(25) $$Bu\underset{T\sim 0}{=}{\left[e^{TU_{T}\left(x^{0}\right)}-\text{Id}\right]}^{-1}U_{T}\left(x^{0}\right)\left(x^{1}-\phi_{T}\left(x^{0}\right)\right)+O\left(T^{2}\right)$$

*where* $U_{T}\left(x^{0}\right):=DN\left(\phi_{T}\left(x^{0}\right)\right)$. *In particular, let* $\tilde{x}(\cdot )$ *denote the solution of* (4) *corresponding to* $\tilde{u}\in {\mathbb{R}}^{k}$ *satisfying*

(26) $$B\tilde{u}={\left[e^{TU_{T}\left(x^{0}\right)}-\text{Id}\right]}^{-1}U_{T}\left(x^{0}\right)\left(x^{1}-\phi_{T}\left(x^{0}\right)\right).$$

*Then, the following endpoint error estimate holds*

$$\left|\tilde{x}(T)-x^{1}\right|\underset{T\sim 0}{=}O\left(T^{2}\right).$$

*Proof.* First, (25) is an immediate consequence of (17), (18) and (24). Next, using the expansions (14), (2), (23), and the definition of $\tilde{u}$ from (26), we obtain

(27) $$\begin{array}{l} \tilde{x}(T)=\phi_{T}\left(x^{0}\right)+\sum_{n=0}^{\infty }{\left(-1\right)}^{n}\frac{T^{n+1}U_{T}{\left(x^{0}\right)}^{n}}{(n+1)!}D\phi_{T}\left(x^{0}\right)B\tilde{u}\\ \;-\varphi_{u}(T)B\tilde{u}\\ \;\underset{T\sim 0}{=}\left[\mathrm{Id}-e^{-TU_{T}\left(x^{0}\right)}\right]U_{T}{\left(x^{0}\right)}^{-1}D\phi_{T}\left(x^{0}\right)B\tilde{u}\\ \;-\left[\mathrm{Id}-e^{-TU_{T}\left(x^{0}\right)}\right]U_{T}{\left(x^{0}\right)}^{-1}D\phi_{T}\left(x^{0}\right)B\tilde{u}\\ \;-\left(e^{TU_{T}\left(x^{0}\right)}-\text{Id}\right)U_{T}{\left(x^{0}\right)}^{-1}B\tilde{u}+\phi_{T}\left(x^{0}\right)\\ \;+O\left(T^{2}\right)\underset{T\sim 0}{=}x^{1}+O\left(T^{2}\right). \end{array}$$

The same observation as in Remark III.9 justifies why the $O\left(T^{3}\right)$ term in (23) reduces to $O\left(T^{2}\right)$ in (27). □

The following result is immediate. It provides a necessary and sufficient condition for (26) to admit at least one solution.

### Corollary III.11.

*Under the hypotheses of Theorem III.7*, (26) *admits at least one solution* $\tilde{u}\in {\mathbb{R}}^{k}$ *if and only if*

$${\left[e^{TU_{T}\left(x^{0}\right)}-\text{Id}\right]}^{-1}U_{T}\left(x^{0}\right)\left(x^{1}-\phi_{T}\left(x^{0}\right)\right)\in \text{Im}\;B.$$

*In this case, the least-norm constant control is given by*

$$\tilde{u}=B^{†}{\left[e^{TU_{T}\left(x^{0}\right)}-\text{Id}\right]}^{-1}U_{T}\left(x^{0}\right)\left(x^{1}-\phi_{T}\left(x^{0}\right)\right).$$

While the previous synthesis applies in a short time, some practical scenarios require reaching the target state over a longer time. In the following, we show how a step function can be constructed from a short-time synthesis to achieve state transfer in a long-time horizon. As noted in Remark II.5, the solution to (4) can still be represented by (9).

We begin with the following implicit synthesis for step-function controls. Its proof, outlined in Section C-C, closely follows the arguments in Proposition III.6 and Theorem III.7.

### Theorem III.12.

*Let* $\left(x^{0},x^{1}\right)\in {\left({\mathbb{R}}^{d}\right)}^{2}$ *and* T≥τ, *where* τ>0 *be such that for any* ℓ∈ℤ, $i\frac{2\pi \ell }{\tau }\notin \sigma \left(DN\left(\phi_{T}\left(x^{0}\right)\right)\right)$. *Define the step-function* $u_{sf,\tau }:[0,T]\to {\mathbb{R}}^{k}$,

$$u_{sf,\tau }(t)=\left\{\begin{array}{ll} 0 & \text{if}\;0\leq t\leq T-\tau \\ u & \text{if}\;T-\tau <t\leq T \end{array}\right.$$

*where* $u\in {\mathbb{R}}^{k}$. *Then, the solution* $x_{\tau }(\cdot )$ *to* (4) *corresponding to* $u_{sf,\tau }(\cdot )$ *satisfies* $x_{\tau }(T)=x^{1}$, *if and only if*, $u\in {\mathbb{R}}^{k}$ *solves*

(28) $$\left[\mathrm{Id}-A_{\tau ,T}\left(x^{0}\right)\varphi_{u}(\tau ,T)\right]Bu=A_{\tau ,T}\left(x^{0}\right)\left(x^{1}-\phi_{T}\left(x^{0}\right)\right).$$

*Here*, $U_{T}\left(x^{0}\right)=DN\left(\phi_{T}\left(x^{0}\right)\right)$ *and*

$$A_{\tau ,T}\left(x^{0}\right)=D\psi_{\tau }\left(\phi_{T}\left(x^{0}\right)\right){\left[\mathrm{Id}-e^{-\tau U_{T}\left(x^{0}\right)}\right]}^{-1}U_{T}\left(x^{0}\right).$$

*Moreover*, $\varphi_{u}(\tau ,T)=\kappa_{u}(\tau ,T)+\eta_{u}(\tau ,T)$, *with* $\kappa_{u}(\tau ,T)$ *and* $\eta_{u}(\tau ,T)$ *defined in* (15) *and* (16), *respectively, where the integrals are taken over* [T−τ,T].

Then, one has the following synthesis in a long-time horizon using explicit step-function controls.

### Theorem III.13.

*Let* $\left(x^{0},x^{1}\right)\in {\left({\mathbb{R}}^{d}\right)}^{2}$ *and* T≥τ, *where* τ>0 *be such that for any* ℓ∈ℤ, $i\frac{2\pi \ell }{\tau }\notin \sigma \left(DN\left(\phi_{T}\left(x^{0}\right)\right)\right)$. *Let* ${\tilde{u}}_{\tau }\in {\mathbb{R}}^{k}$ *be a solution of*

$$B{\tilde{u}}_{\tau }={\left[e^{\tau U_{T}\left(x^{0}\right)}-\text{Id}\right]}^{-1}U_{T}\left(x^{0}\right)\left(x^{1}-\phi_{T}\left(x^{0}\right)\right)$$

*where* $U_{T}\left(x^{0}\right):=DN\left(\phi_{T}\left(x^{0}\right)\right)$. *Then, the solution* ${\tilde{x}}_{\tau }(\cdot )$ *to* (4) *corresponding to the step function* ${\tilde{u}}_{sf,\tau }:[0,T]\to {\mathbb{R}}^{k}$,

$${\tilde{u}}_{sf,\tau }(t)=\left\{\begin{array}{ll} 0 & \text{if}\;0\leq t\leq T-\tau \\ {\tilde{u}}_{\tau } & \text{if}\;T-\tau <t\leq T \end{array}\right.$$

*satisfies*

$$\left|{\tilde{x}}_{\tau }(T)-x^{1}\right|\underset{\tau \sim 0}{=}O\left(\tau^{2}\right).$$

The proof of Theorem III.13 follows the same arguments as Theorem III.10 and is therefore omitted for brevity.

#### 2) Backward nominal-state synthesis:

Building on the solution representation of (4) given in Theorem II.4, we aim to construct step-function controls under the assumption that the terminal condition $x(T)=x^{1}$ is achieved. In contrast to the forward nominal-state synthesis, the implicit control derived here does not provide a direct condition guaranteeing that the target state is reached.

We begin with the key result on which the implicit synthesis is based. The proof is presented in Section C-A.

### Proposition III.14.

*Let* T>0 *and* $\left(x_{0},u\right)\in {\mathbb{R}}^{d}\times {\mathbb{R}}^{k}$. *The solution* x(⋅) *of* (4) *can be expanded as*

(29) $$x(t)=\phi_{t}\left(x^{0}+\sum_{n=0}^{\infty }\frac{t^{n+1}Z_{t}^{n}}{(n+1)!}P_{t}Bu-\zeta_{u}(t)Bu\right)$$

*for all* t∈[0,T]. *Here* $\zeta_{u}(t)=\xi_{u}(t)+\chi_{u}(t)$,

(30) $$\xi_{u}(t)=\sum_{n=1}^{\infty }\int_{0}^{t}\frac{s^{n+1}}{(n+1)!}\left[\frac{d}{ds}Z_{s}^{n}\right]P_{s}ds,$$

(31) $$\chi_{u}(t)=\sum_{n=1}^{\infty }\int_{0}^{t}\frac{s^{n}}{n!}Z_{s}^{n-1}D^{2}\psi_{s}(x(s))\dot{x}(s)ds,$$

*where* $P_{t}=D\psi_{t}(x(t))$, $Z_{t}=DN\left(\psi_{t}(x(t))\right)$ *and* $D^{2}\psi_{s}(x(s))$ *is the second derivative of* $\psi_{s}$ *at* x(s).

### Theorem III.15.

*Let* T>0 *and* $\left(x^{0},x^{1}\right)\in {\left({\mathbb{R}}^{d}\right)}^{2}$. *Assume that for any* ℓ∈ℤ, $i\frac{2\pi \ell }{T}\notin \sigma \left(DN\left(\psi_{T}\left(x^{1}\right)\right)\right)$. *Any constant control* $u\in {\mathbb{R}}^{k}$ *that ensures the corresponding solution* x(⋅) *of* (4) *satisfies* $x(T)=x^{1}$ *necessarily solve*

(32) $$\left(\mathrm{Id}-B_{T}\left(x^{1}\right)\zeta_{u}(T)\right)Bu=B_{T}\left(x^{1}\right)\left(\psi_{T}\left(x^{1}\right)-x^{0}\right).$$

*Here* $\zeta_{u}(T)\in M_{d}(\mathbb{R})$ *is defined in Proposition III.14, and letting* $V_{T}\left(x^{1}\right)=DN\left(\psi_{T}\left(x^{1}\right)\right)$, *one has*

(33) $$B_{T}\left(x^{1}\right)=D\phi_{T}\left(\psi_{T}\left(x^{1}\right)\right){\left(e^{TV_{T}\left(x^{1}\right)}-\text{Id}\right)}^{-1}V_{T}\left(x^{1}\right).$$

### Remark III.16.

The implicit backward nominal-state synthesis in Theorem III.15 provides only a necessary condition for a constant control $u\in {\mathbb{R}}^{k}$ to achieve $x(T)=x^{1}$ in (4). In contrast, the forward synthesis (17) yields a sufficient condition: any u solving it guarantees $x(T)=x^{1}$, and thus also satisfies the backward condition.

Let us now present the proof of Theorem III.15.

*Proof of* Theorem III.15. Letting t=T in (29), one gets from $x(T)=x^{1}$ and left multiplication by $V_{T}\left(x^{1}\right)$ that

(34) $$V_{T}\left(x^{1}\right)\left(\psi_{T}\left(x^{1}\right)-x^{0}\right)=-V_{T}\left(x^{1}\right)\zeta_{u}(T)Bu\;\;+\left(e^{TV_{T}\left(x^{1}\right)}-\text{Id}\right)D\psi_{T}\left(x^{1}\right)Bu$$

by (2). Since ${\left[D\psi_{T}\left(x^{1}\right)\right]}^{-1}=D\phi_{T}\left(\psi_{T}\left(x^{1}\right)\right)$, left multiplying (34) by ${\left(e^{TV_{T}\left(x^{1}\right)}-\text{Id}\right)}^{-1}$ and $D\phi_{T}\left(\psi_{T}\left(x^{1}\right)\right)$ yields

$$\begin{array}{l} D\phi_{T}\left(\psi_{T}\left(x^{1}\right)\right){\left(e^{TV_{T}\left(x^{1}\right)}-\text{Id}\right)}^{-1}V_{T}\left(x^{1}\right)\left(\psi_{T}\left(x^{1}\right)-x^{0}\right)=\\ \;Bu-D\phi_{T}\left(\psi_{T}\left(x^{1}\right)\right){\left(e^{TV_{T}\left(x^{1}\right)}-\text{Id}\right)}^{-1}V_{T}\left(x^{1}\right)\zeta_{u}(T)Bu. \end{array}$$

It follows that $u\in {\mathbb{R}}^{k}$ solves the implicit equation

$$\left[\text{Id}-B_{T}\left(x^{1}\right)\zeta_{u}(T)\right]Bu=B_{T}\left(x^{1}\right)\left(\psi_{T}\left(x^{1}\right)-x^{0}\right)$$

which is (32), where $B_{T}\left(x^{1}\right)$ is defined by (33). □

Using the same machinery that leads to Theorem III.10, we obtain the following result. The proof is omitted for brevity.

### Theorem III.17.

*Under the assumptions of Theorem III.15, let* $\bar{x}(\cdot )$ *denote the solution of* (4) *corresponding to a constant control* $\bar{u}\in {\mathbb{R}}^{k}$ *satisfying*

(35) $$B\bar{u}={\left[\mathrm{Id}-e^{-TV_{T}\left(x^{1}\right)}\right]}^{-1}V_{T}\left(x^{1}\right)\left(\psi_{T}\left(x^{1}\right)-x^{0}\right)$$

*where* $V_{T}\left(x^{1}\right):=DN\left(\psi_{T}\left(x^{1}\right)\right)$. *Then, the following endpoint error estimate holds*

$$\left|\bar{x}(T)-x^{1}\right|\underset{T\sim 0}{=}O\left(T^{2}\right).$$

The following result provides a necessary and sufficient condition for (35) to admit at least one solution.

### Corollary III.18.

*Under the hypotheses of Theorem III.15*, (35) *admits at least one solution* $\tilde{u}\in {\mathbb{R}}^{k}$ *if and only if*

$${\left[\mathrm{Id}-e^{-TV_{T}\left(x^{1}\right)}\right]}^{-1}V_{T}\left(x^{1}\right)\left(\psi_{T}\left(x^{1}\right)-x^{0}\right)\in \text{Im}\;B.$$

*In this case, the least-norm constant control is given by*

$$\bar{u}=B^{†}{\left[\mathrm{Id}-e^{-TV_{T}\left(x^{1}\right)}\right]}^{-1}V_{T}\left(x^{1}\right)\left(\psi_{T}\left(x^{1}\right)-x^{0}\right).$$

Finally, one has the following result that synthesizes a step function for a large time horizon.

### Theorem III.19.

*Let* $\left(x^{0},x^{1}\right)\in {\left({\mathbb{R}}^{d}\right)}^{2}$ *and* T≥τ, *where* τ>0 *be such that for any* ℓ∈ℤ, $i\frac{2\pi \ell }{\tau }\notin \sigma \left(DN\left(\psi_{T}\left(x^{1}\right)\right)\right)$. *Let* ${\bar{u}}_{\tau }\in {\mathbb{R}}^{k}$ *be a solution of*

$$B{\bar{u}}_{\tau }={\left[\mathrm{Id}-e^{-\tau V_{T}\left(x^{1}\right)}\right]}^{-1}V_{T}\left(x^{1}\right)\left(\psi_{T}\left(x^{1}\right)-x^{0}\right)$$

*where* $V_{T}\left(x^{1}\right)=DN\left(\psi_{T}\left(x^{1}\right)\right)$. *Then, the solution* x(⋅) *to* (4) *corresponding to the step function* ${\bar{u}}_{sf}:[0,T]\to {\mathbb{R}}^{k}$,

$${\bar{u}}_{sf}(t)=\left\{\begin{array}{ll} 0 & if\;0\leq t\leq T-\tau \\ \bar{u} & if\;T-\tau <t\leq T \end{array}\right.$$

*satisfies*

$$\left|x(T)-x^{1}\right|\underset{\tau \sim 0}{=}O\left(\tau^{2}\right).$$

### Remark III.20.

A key distinction between the forward and backward nominal-state syntheses lies in their respective spectral conditions: the forward synthesis depends on the spectrum at the initial state $x^{0}$, while the backward synthesis relies on that at the target state $x^{1}$.

In practice, only one of these conditions may be satisfied, depending on the region of the state space. This asymmetry underscores the *complementarity* of the two syntheses—each offers a valid control strategy under different local spectral properties, making their coexistence practically valuable.

This contrast also reflects a deeper difference between linear and nonlinear control. In the linear case, controllability depends on a uniform spectral condition (cf. Proposition III.2); failure at one point implies failure everywhere. In the nonlinear setting, local spectral conditions at either the initial or target can independently ensure controllability, highlighting a key advantage of the nonlinear synthesis framework.

#### C. Forward and Backward syntheses

Although Sections III-B1 and III-B2 focus on the *forward* and *backward nominal-state* syntheses, respectively, it is worth emphasizing that both arise from a broader family of synthesis strategies derived from the dual representations of the nonlinear system (4) in Theorems II.2 and II.4. Each representation admits at least two distinct expansion strategies—depending on the chosen integration by parts identity—that lead to meaningful control synthesis formulations.

Specifically, starting from the solution representation (7), we observe two synthesis paths:

- Applying the identity $\int_{0}^{T}D\psi_{t}dt=\int_{0}^{T}t^{\prime }D\psi_{t}dt$ yields the implicit *backward nominal-state* synthesis fully investigated in Section III-B2.
- Using instead $\int_{0}^{T}D\psi_{t}dt=\int_{0}^{T}{\left(t-T\right)}^{\prime }D\psi_{t}dt$ gives rise to what we refer to as an implicit *backward initial-state* synthesis. This formulation yields an expression of the control that provides a necessary and sufficient condition for reachability, and it is not analyzed in detail here.

Similarly, from the solution representation (9), two analogous options arise:

- Using the identity $\int_{0}^{T}D\phi_{T-t}dt=\int_{0}^{T}{\left(t-T\right)}^{\prime }D\phi_{T-t}dt$ leads to the implict *forward nominal-state* synthesis fully developed in Section III-B1.
- Alternatively, using $\int_{0}^{T}D\phi_{T-t}dt=\int_{0}^{T}t^{\prime }D\phi_{T-t}dt$ results in what we refer to as an implicit *forward final-state synthesis*. This yields a formally valid expansion and a necessary condition on the control, and it is not investigated in this work.

#### D. Reachability via control synthesis methods

In this section, we summarize the main results from Sections III-B1 and III-B2 into a set of operational insights framed in terms of the input matrix B. Specifically, we analyze how the set of states reachable from a given initial condition $x^{0}\in {\mathbb{R}}^{d}$ over the interval [0,T] depends on the structure of B, under both constant and step-function control strategies synthesized via the proposed methods.

Given $x^{0}\in {\mathbb{R}}^{d}$, we say that a state $x^{1}\in {\mathbb{R}}^{d}$ is *c-reachable over* [0,T] from $x^{0}$ if there exists a constant control $u\in {\mathbb{R}}^{k}$ such that the solution x(⋅) of (4) corresponding to u satisfies $x(T)=x^{1}$. The c-reachable set is denoted by (for step-function controls, we denote it by $R_{sc}\left(\tau ,T,x^{0}\right)$)

(36) $$R_{c}\left(T,x^{0}\right)=\left\{x(T)\left|\begin{array}{l} x(\cdot )\;\text{solves (4)}\\ \text{with constant control}\;u\in {\mathbb{R}}^{k} \end{array}\right.\right\}.$$

We say that (4) is *controllable over* [0,T] from $x^{0}$ with a constant control if $R_{c}\left(T,x^{0}\right)={\mathbb{R}}^{d}$. If this holds for every $x^{0}\in {\mathbb{R}}^{d}$, then (4) is said to be *completely controllable* over [0,T] with constant controls.

##### 1) The linear case:

It is convenient to begin with the linear system (10), which helps understanding the fully nonlinear setting. In the linear control framework, controllability with a time-varying control is guaranteed if the Kalman rank condition or, equivalently, the invertibility of the controllability Gramian holds. These conditions are independent of T>0 and $x^{0}$, and they allow for flexibility in choosing the input matrix $B\in M_{d,k}(\mathbb{R})$ satisfying what we refer to as the *time-varying controllability condition*.

However, as shown in Remark III.1, the question becomes more delicate when restricting to constant controls. Notably, even the trivial case B=Id may fail to achieve controllability with a constant control, despite satisfying the time-varying controllability condition.

Under the spectral condition in Proposition III.2, Corollary III.3 shows that (10) is *completely controllable with constant controls* over [0,T] if and only if k=d and B is invertible. Otherwise, if k<d or B is not invertible, then

$$R_{c}\left(T,x^{0}\right)⊈{\mathbb{R}}^{d},\;\text{for all}\;x^{0}\in {\mathbb{R}}^{d}.$$

Hence, the size of $R_{c}\left(T,x^{0}\right)$ is determined by the rank of B: the smaller k is relative to d, the fewer the number of target states that are reachable from $x^{0}$ via constant control.

##### 2) The Nonlinear Case:

We now turn to the fully nonlinear setting and analyze the c-reachable set $R_{c}\left(T,x^{0}\right)$, leveraging the forward nominal-state synthesis from Section III-B1.

Unlike the linear case, the synthesis equation in Theorem III.7 is implicit in u unless $\varphi_{u}(T)=0$. When $\varphi_{u}(T)=0$, the analysis from Section III-D1 directly applies. If $\varphi_{u}(T)\neq 0$, Proposition III.8 shows that for sufficiently small T>0, the control u satisfies (25). As a result, (4) is *completely controllable with constant controls* over [0,T] for small T>0 if and only if k=d and B is invertible. Otherwise, when k<d or B is not invertible, the c-reachable set satisfies

$$R_{c}\left(T,x^{0}\right)⊈{\mathbb{R}}^{d},\;\text{for all}\;x^{0}\in {\mathbb{R}}^{d},$$

and its size is again governed by the rank of B.

Still, when $\varphi_{u}(T)\neq 0$, the small-time reachability result allows one to extend the synthesis to arbitrary horizons T>0 via *step-function controls*. Specifically, let τ>0 be such that for any ℓ∈ℤ, $\frac{2\pi \ell }{\tau }\notin \sigma \left(DN\left(\phi_{T}\left(x^{0}\right)\right)\right.$. Then, for T≤τ, we have $R_{sc}\left(\tau ,T,x^{0}\right)=R_{c}\left(\tau ,x^{0}\right)$. While for T>τ, Theorem III.12 shows that system (4) is *completely controllable with step-function controls* over [0,T] if and only if k=d and B is invertible. Otherwise, one has

$$R_{sc}\left(\tau ,T,x^{0}\right)⊊{\mathbb{R}}^{d},\;\text{for all}\;x^{0}\in {\mathbb{R}}^{d}$$

and the sc-reachable set structure again depends on rank(B).

Let us characterize $R_{c}\left(T,x^{0}\right)$ for the input matrix traditionally considered in network neuroscience [16], namely

(37) $$B=\left[\begin{array}{llll} e_{1} & e_{2} & \ldots & e_{k} \end{array}\right],\;k<d,$$

where $e_{i}$ is the i–th canonical basis vector of ${\mathbb{R}}^{d}$.

### Proposition III.21.

*Let* T>0 *and* $\left(x^{0},x^{1}\right)\in {\left({\mathbb{R}}^{d}\right)}^{2}$. *Assume that for any* ℓ∈ℤ, $i\frac{2\pi \ell }{T}\notin \sigma \left(U_{T}\left(x^{0}\right)\right)$. *If the input matrix* B *is given by* (37), *then the c-reachable set satisfies*

(38) $$\begin{array}{l} R_{c}\left(T,x^{0}\right)\underset{T\sim 0}{=}\phi_{T}\left(x^{0}\right)+\ker M_{T}\left(x^{0}\right)+O\left(T^{2}\right),\\ \;\dim\left(\ker M_{T}\left(x^{0}\right)\right)=k, \end{array}$$

*and the constant input* $u\in {\mathbb{R}}^{k}$ *that steers* (4) *from* $x^{0}\in {\mathbb{R}}^{d}$ *to* $x^{1}\in \phi_{T}\left(x^{0}\right)+\ker M_{T}\left(x^{0}\right)$ *is given by*

(39) $$u=V_{T}\left(x^{0}\right)\left(x^{1}-\phi_{T}\left(x^{0}\right)\right).$$

*Here* $U_{T}\left(x^{0}\right):=DN\left(\phi_{T}\left(x^{0}\right)\right)$, $V_{T}\left(x^{0}\right)\in M_{k,d}(\mathbb{R})$ *collects the* k *nonzero rows of* $B_{T}\left(x^{0}\right):={\left[e^{TU_{T}\left(x^{0}\right)}-\text{Id}\right]}^{-1}U_{T}\left(x^{0}\right)$, *and* $M_{T}\left(x^{0}\right)\in M_{d-k,d}(\mathbb{R})$ *collects its* d−k *zero rows*.

*Proof.* First, it follows from Theorem III.10 that the solution x(⋅) to (4) corresponding to a constant control $u\in {\mathbb{R}}^{k}$ solving

(40) $$Bu={\left[e^{TU_{T}\left(x^{0}\right)}-\text{Id}\right]}^{-1}U_{T}\left(x^{0}\right)\left(x^{1}-\phi_{T}\left(x^{0}\right)\right)$$

satisfies (see (27))

(41) $$x(T)\underset{T∼0}{=}x^{1}+O\left(T^{2}\right).$$

Next, with B as in (37), one deduces from (40) that

(42) $$\left[\begin{array}{c} {\text{Id}}_{k}\\ O_{d-k,k} \end{array}\right]u=\left[\begin{array}{c} V_{T}\left(x^{0}\right)\\ M_{T}\left(x^{0}\right) \end{array}\right]\left(x^{1}-\phi_{T}\left(x^{0}\right)\right)$$

where $\text{I}{\text{d}}_{k}\in M_{k}(\mathbb{R})$ is the identity matrix, and $O_{d-k,k}\in M_{d-k,k}(\mathbb{R})$ is the zero matrix. Since $B_{T}\left(x^{0}\right)$ is invertible, one has rank $M_{T}\left(x^{0}\right)=d-k$ and therefore $\dim\left(\ker M_{T}\left(x^{0}\right)\right)=d-(d-k)=k$. Finally, (42) is equivalent to (39) and

(43) $$x^{1}\in \phi_{T}\left(x^{0}\right)+\ker M_{T}\left(x^{0}\right).$$

Combining (36), (41) and (43) yields (38). □

### Remark III.22.

In Proposition III.21, the first-order characterization of the c-reachable set as an affine subspace is valid as long as the desired target shift $x^{1}-\phi_{T}\left(x^{0}\right)=O(T)$. This ensures that the required constant input (39) remains bounded, since to leading order T→0, one has $|u|\approx \left|x^{1}-\phi_{T}\left(x^{0}\right)\right|/T$.

Using the thin QR factorization of $M_{T}{\left(x^{0}\right)}^{⊤}\in {\mathbb{R}}^{d\times (d-k)}$,

$$M_{T}{\left(x^{0}\right)}^{⊤}=\left[\begin{array}{ll} Q_{1} & Q_{2} \end{array}\right]\left[\begin{array}{c} R_{1}\\ 0 \end{array}\right],\;\left\{\begin{array}{l} Q_{1}\in {\mathbb{R}}^{d\times (d-k)},Q_{2}\in {\mathbb{R}}^{d\times k},\\ R_{1}\in {\mathbb{R}}^{\left(d-k)\times (d-k\right)}, \end{array}\right.$$

one finds that ker $M_{T}\left(x^{0}\right)=\mathrm{span}\left(Q_{2}\right)$.

### Remark III.23.

First, the c-reachable set characterization and the associated constant input synthesis in Proposition III.21 for a small time horizon T>0 when the input matrix B is given by (37) can be improved by explicitly considering the second derivative $D^{2}\phi_{T-t}$ in the expansion. We defer this analysis to future works. Next, let T>0 and $x^{0}\in {\mathbb{R}}^{d}$ be such that $DN\left(\phi_{T}\left(x^{0}\right)\right)$ satisfies the spectral condition in Theorem III.7. Future work should investigate the structure and effective dimension of the c-reachable sets $R_{c}\left(T,x^{0}\right)$ as functions of $\text{rank}\left(B\right)$ for a general input matrix $B\in M_{k,d}(\mathbb{R})$.

## IV. Comments on the main results of Section III

First, if the spectral norm condition $\left\|W\|<\lambda_{\min}(D\right)$ is satisfied, then the eigenvalue-related assumptions required in Proposition III.2,Theorems III.7, and III.15 are automatically fulfilled, as discussed, e.g., in Remark III.4. Note that this condition ensures that the nonlinear system (4) is contracting.

Contracting dynamics possess a range of desirable properties–see [20]–and contraction has become a standard structural assumption in many recent works on recurrent neural networks, such as [7], [11]. While the condition on ‖W‖ is a sufficient criterion for contraction, it serves here as a simple and practically verifiable condition that ensures well-posedness of the synthesis proposed in this work.

More generally, the control syntheses developed in this paper apply to any nonlinear system of the form $\dot{x}(t)=N(x(t))+Bu$, where $u\in {\mathbb{R}}^{k}$ is constant and $N\in C^{2}\left({\mathbb{R}}^{d};{\mathbb{R}}^{d}\right)$ satisfies the regularity conditions

$$\underset{x\in {\mathbb{R}}^{d}}{\sup}\|DN(x)\|\leq C,\;\underset{x\in {\mathbb{R}}^{d}}{\sup}\|D^{2}N(x)\|\leq C$$

for some constant C>0. Notably, we do not require any explicit boundedness condition on the vector field N itself, making the framework broadly applicable.

This flexibility is particularly relevant for applications in machine learning, where recurrent neural networks (RNNs) often employ unbounded and non-smooth activation functions such as the $\mathrm{ReLU}:f_{i}\left(x_{i}\right)=\mathrm{ReLU}\left(x_{i}\right)=\max\left(0,x_{i}\right)$. ReLU is differentiable everywhere except at $x_{i}=0$, and it is globally Lipschitz on ℝ. In this case, the solution representations (7) and (9) no longer apply, and our control syntheses cannot be directly used. To overcome this limitation, one can consider smooth approximations of the ReLU function (e.g., softplus [14], swish function [29]), which restore differentiability and allow the application of our synthesis results.

## V. Examples and numerical simulations

**Fig. 2.**
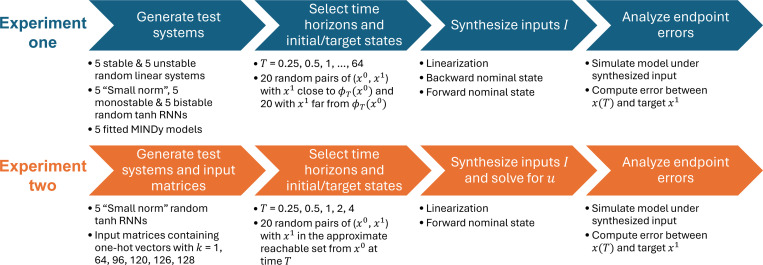
Pipeline of the numerical experiments.

This section presents some numerical simulations that underpin our theoretical study. The pipeline of the experiments and analysis is visualized in Fig. 2. We included three major types of models: linear systems, “vanilla” tanh RNNs, and MINDy models [32]. While vanilla RNNs are widely used in theoretical neuroscience as task-performing models, MINDy models represent another branch of models that directly approximate experimental neural data, demonstrating the different applicative contexts of our theoretical framework.

### A. Implementation

We implement the linear system control synthesis (13), the nonlinear forward nominal-state synthesis (26), and the nonlinear backward nominal-state synthesis (35) in python. The scripts will be available after publication. For the nonlinear synthesis, the flow is numerically integrated using the odeint function from the torchdiffeq package [9]. We adopted the Runge-Kutta method of order 7(8) of Dormand-Prince-Shampine, which is the highest-order method available from the package. The method accepts a new step if $\text{RMS}(\varepsilon )<10^{-14}+10^{-13}*\text{RMS}(x)$, where RMS(⋅) represents the root mean square norm, ε being the estimated error and x the current state. We found that this setting provided reasonable control of the numerical error related to flow integration. The Jacobian matrices were computed through auto-differentiation in pytorch. Experiments were conducted on a desktop computer with an Nvidia RTX 3080 GPU. The run time for each synthesis increased as the time horizon T and number of features (neurons) d increased, but remained within the scope of several hundred milliseconds to several seconds. For a fixed T and multiple desired trajectories (i.e., different pairs of $\left(x^{0},x^{1}\right)$), the synthesis is efficiently parallelized, and the run time remains almost constant when increasing the number of trajectories from one to 1000.

To construct a d-dimensional linear system, we randomly sampled the entries of W from the normal distribution N(0,1/d). The decay matrix was set to $\left(\lambda_{\max}(W)-\lambda_{0}\right)\text{Id}$, where $\lambda_{\max}(W)\in \mathbb{R}$ is the maximum of the real parts of the eigenvalues of W and $\lambda_{0}\in \mathbb{R}$ is the desired maximum real part of the eigenvalues of the dynamic matrix A=W−D. We tried both $\lambda_{0}=-0.1$ and $\lambda_{0}=0.1$ to construct stable and unstable systems respectively.

To construct a “vanilla” RNN, we set D=Id and the activation function to tanh. We followed [35] and imposed a “random plus low rank” structure on the connectivity matrix $W=J+mn^{⊤}$. The matrix $J\in M_{d}(\mathbb{R})$ was sampled from the normal distribution $N\left(0,g^{2}/d\right)$, where g is a scaling factor. m, $n\in M_{d,p}(\mathbb{R})$ determines the rank of the low-rank component. Such a type of model appears frequently in theoretical neuroscience studies [23]. We considered three subtypes of vanilla RNNs. The first subtype is referred to as ‘small norm tanh RNN’, constructed with g=0.5 and p=0 (no low-rank component). We found that such RNNs satisfied $\left\|WDf(0)\|\approx 1=\lambda_{\min}(D\right)$. For the next two subtypes, g was set to 0.9 [35] and the spectral norm condition was violated. $m\in {\mathbb{R}}^{d}$ was sampled from standard normal distribution. $n\in {\mathbb{R}}^{d}$ was sampled from normal distribution $N\left(0,1/d^{2}\right)$ for the second subtype and set as $n=\frac{1.1}{d}m$ for the third subtype. It can be shown that systems of the second subtype will be monostable while systems of the third subtype will be bistable [35] as the number of neurons d tends to infinity.

To further evaluate the method on realistic models fit to *experimental data*, we also included a set of Mesoscale Individualized Neurodynamic (MINDy) models from [32]. MINDy models contained 100 interconnected units representing 100 brain areas, and the parameters were optimized to approximate the activation time series of these areas measured through functional magnetic resonance imaging (fMRI). Unlike most RNNs, the activation function of MINDy is heterogeneous: $f(s)=\sqrt{\alpha^{2}+{\left(bs+0.5\right)}^{2}}-\sqrt{\alpha^{2}+{\left(bs-0.5\right)}^{2}}$, where b=20/3 is fixed and α is optimized over the data and differs across the 100 units. It is also worth noting that the origin is unstable in most of the MINDy models, indicating that the spectral norm condition $\left\|WDf(0)\|<\lambda_{\min}(D\right)$ was not met. However, we found that the models satisfy the eigenvalue condition, which enabled the synthesis.

In the first experiment, we analyzed the endpoint error of the controlled trajectory for $T\in \left\{2^{-2},2^{-1},\ldots ,2^{6}\right\}$ when B=Id. For each T, we randomly generated 5 stable and 5 unstable linear systems, 5 small-norm, 5 monostable and 5 bistable tanh RNNs, and randomly selected 5 fitted MINDy models (from a pool of 106 models). All models were 100-dimensional. Then, we randomly generated 40 initial states $x^{0}\sim N(0,\text{Id})$. To investigate how the deviation of $x^{1}$ from the autonomous flow end point $\phi_{T}\left(x^{0}\right)$ influences the performance of the method, we set $x^{1}=\phi_{T}\left(x^{0}\right)+\varepsilon$ where $\varepsilon \sim N\left(0,\sigma^{2}\cdot \text{Id}\right)$. In half of the trials, $\sigma^{2}$ was set to 0.1 (“small deviation”); in the other half, $\sigma^{2}$ was set to 0.5 (“large deviation”). Control input was computed using the nonlinear forward (26) and backward (35) syntheses, and the linear synthesis (13). We recall that for the nonlinear system (4), when B=Id, the linearized system at $x^{0}$ is given by

$$\dot{x}(t)=DN\left(x^{0}\right)x(t)+N\left(x^{0}\right)+u.$$

Using (13), the following input u drives the linearized system from $x^{0}$ to $x^{1}$ in exact time T

(44) $$u={\left(e^{TA}-\text{Id}\right)}^{-1}A\left(x^{1}-e^{TA}x^{0}\right)-N\left(x^{0}\right)$$

where $A=DN\left(x^{0}\right)$. We have also tried to linearize the systems at the origin (which is always a fixed point) and obtained qualitatively similar results.

In the second experiment, we followed the tradition in network neuroscience [16] and considered the input matrix defined by (37). For simplicity, we focused on 128dimensional “small norm” tanh RNNs (with g=0.5 and p=0) and T∈{0.25,0.5,1,2,4}. We considered k∈{1,64,96,120,126,128}, ranging from an extremely underactuated system to a fully actuated system. We randomly generated 5 RNNs and selected 20 initial states $x^{0}\sim N(0,\text{Id})$ for each model and each combination of T and k. Here, $x^{1}=\phi_{T}\left(x^{0}\right)+Q_{2}\xi$ by Proposition III.21, and $Q_{2}$ following Remark III.22, with entries of ξ drawn from N(0,0.01).

### B. Results

**Fig. 3.**
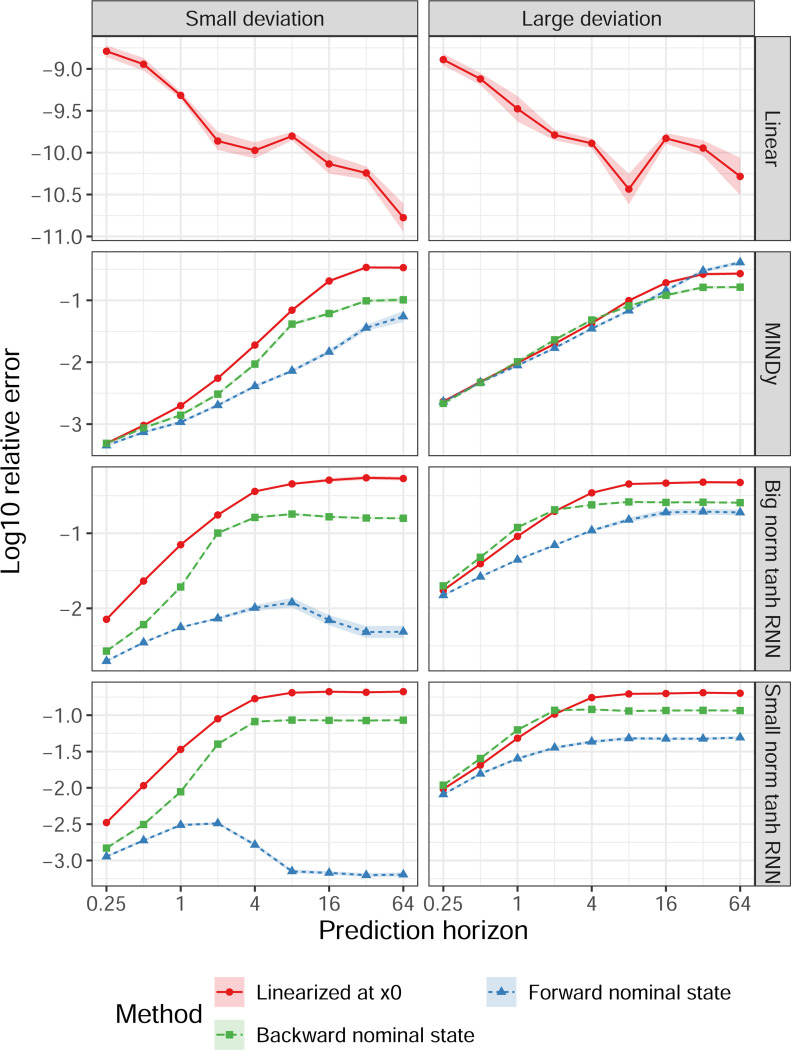
Relative endpoint error under synthesized input. The x-axis (log scale) indicates the time horizon T. The y-axis represents the common logarithm of the ratio between the Euclidean norm of the endpoint error $x(T)-x^{1}$ and the Euclidean norm of $x^{1}-x^{0}$. Results were organized according to the combination of model type (rows) and how $x^{1}$ were specified (columns). Monostable and bistable RNNs were merged into a single category of “Big norm tanh RNN” due to similarity. Results using linearization (44), nonlinear forward nominal-state synthesis (26), and nonlinear backward nominal-state synthesis (35) were shown in red, blue and green respectively. Line plots and error bands represent the mean and 95% confidence interval of log relative error.

Results of the first experiment were summarized in Fig. 3. Monostable and bistable tanh RNNs were combined into “Big norm tanh RNN” due to similarity. For linear systems, the error remained small. For nonlinear systems, error generally increased as the horizon T increased. The forward nominal state synthesis performed better than the backward nominal state synthesis, and both of them generally performed better than linearization, particularly for large T. The differences were more evident when $x^{1}-\phi_{T}\left(x^{0}\right)$ is small (with standard deviation 0.1, left panels), where the forward synthesis sometimes outperformed linearization by orders of magnitude. Note that the MINDy models were trained to approximate timeseries with unit variance, so a “small deviation” with standard deviation 0.1 already represents a physically sizable difference, indicating the practical significance of our method.

**Fig. 4.**
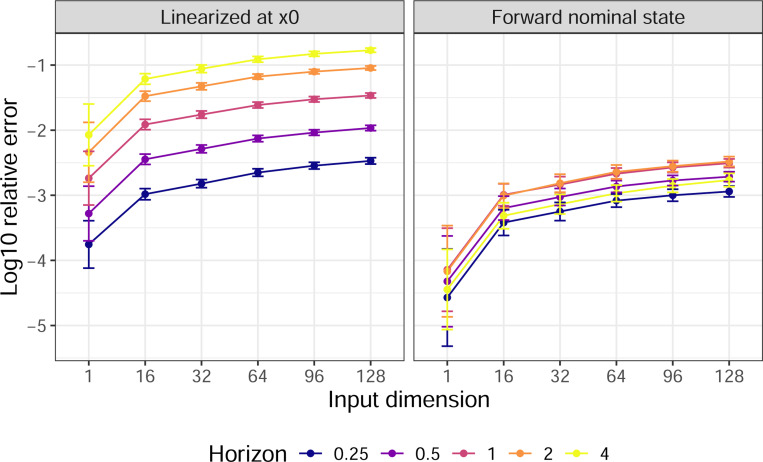
Relative endpoint error under synthesized input for underactuated systems. We considered “small norm tanh RNNs” of 128 dimension. The x-axis (ordinal scale) indicates the input dimension k. The y-axis represents the common logarithm of the relative endpoint error. Results using linearized and nonlinear forward synthesis methods were separated into the two panels. Color indicates different time horizons T. Line plots and error bars represent the mean and standard deviation of log relative error over 20 experiments.

Results of the second experiment were summarized in Fig. 4. The nonlinear synthesis was still much better than linearization in underactuated systems, as expected. However, surprisingly, the endpoint error did not change dramatically and even decreased as the number of actuators decreased, as long as $x^{1}$ is close to the reachable set. These findings suggest that Proposition III.21 did provide a good estimation of the reachable set, and that the synthesis worked in underactuated systems just as good as (if not better than) fully-actuated systems as long as $x^{1}$ is reachable.

## VI. Concluding remarks and perspectives

This paper addressed the control synthesis problem for a class of nonlinear Hopfield-type recurrent neural networks motivated by neurostimulation applications. Using a solution representation that generalizes the variation of constants formula, we derived constant and piecewise constant inputs capable of steering the network to a desired target within a prescribed time interval. For linear activation functions, the exact controllability with constant input is possible over arbitrary time horizons. For a small time horizon, we also provided a characterization of the reachable set when the input matrix B directly actuates a subset of nodes, showing that its dimension equals the rank of B, and that its basis can be computed efficiently via a thin QR factorization. Moreover, the constant input that guarantees reachability is given by a closed-form algebraic condition, which makes the synthesis directly applicable for tDCS.

Future work will explore the synthesis of time-varying control inputs based on the proposed framework, with an emphasis on robustness to parameter uncertainty and external disturbances. As large-scale systems like (4) are highly sensitive to such perturbations, designing controls that adapt dynamically while minimizing energy costs remains a key challenge—one that links controllability with energetic performance.

## Supplementary Material

Supplement 1
