# Supplementary material for: On the control of recurrent neural networks using constant inputs
Source: ArXiv. 2025 Sep 26:arXiv:2410.17199v2. Originally published 2024 Oct 22. Preprint. [Version 2] (PMC11537334)
Supplement: Supplement 1 [file NIHPP2410.17199v2-supplement-1.pdf]

Appendix A
General results on the drift and its associated flow maps

This section contains various supplementary results related to the vector field N that were used intensively in the previous sections. The first result concerns some useful properties of the Fréchet-differential DN and the second-differential D^2N.

Lemma A.1. *The vector field N defined in (3) belongs to $C^2(\mathbb{R}^d;\mathbb{R}^d)$, and it is Lipschitz continuous from $\mathbb{R}^d$ into itself. Moreover, the following hold*

$$\begin{aligned} DN(x) &= -D + WDf(x) \\ \|DN(x)\| &\leq \lambda_{\max}(D) + \|W\| \end{aligned} \quad \forall x \in \mathbb{R}^d. \quad (45)$$

$$\begin{aligned} D^2N(x) &= WD^2f(x) \\ \|D^2N(x)\| &\leq \|f''\|_\infty \|W\| \end{aligned} \quad \forall x \in \mathbb{R}^d. \quad (46)$$

Here $\|f''\|_\infty = \max_{i\in[\![1,d]\!]} \|f_i''\|_\infty$, $Df(x) = (f_i'(x_i)\delta_{i,j})$ and $D^2f(x)$ is a third order tensor such that $D^2f(x)yz \in \mathbb{R}^d$ for all y, $z \in \mathbb{R}^d$, and the i-th element is given, by

$$[D^2f(x)yz]_{|i} = \sum_{j,k=1}^{d} \frac{\partial^2 f_i}{\partial x_j \partial x_k}(x) y_j z_k = f_i''(x_i) y_i z_i. \quad (47)$$

Proof. Let $x, y \in \mathbb{R}^d$. Since $|\!|\!|Df(x)|\!|\!| \leq 1$, one has

$$|f(x) - f(y)| \leq \|Df(x)\| |x - y| \leq |x - y|.$$

One deduces that

$$|N(x) - N(y)| \leq (\lambda_{\max}(D) + \|W\|)|x - y|$$

showing that N is globally Lipschitz continuous from $\mathbb{R}^d$ into itself. On one hand, one has obviously that $N \in C^2(\mathbb{R}^d;\mathbb{R}^d)$ since $f \in C^2(\mathbb{R}^d;\mathbb{R}^d)$ by assumption. It follows that $DN(x)h = -Dh + WDf(x)h$ is linear w.r.t. $h \in \mathbb{R}^d$ and satisfies

$$|DN(x)h| \leq (\lambda_{\max}(D) + \|W\|)|h|$$

so that (45) is satisfied. On the other hand, for all y, $z \in \mathbb{R}^d$, one has

$$D^2N(x)yz = WD^2f(x)yz.$$

Since $f : \mathbb{R}^d \to \mathbb{R}^d$, $D^2f(x)$ is a third order tensor, one has $D^2f(x)yz \in \mathbb{R}^d$ for all y, $z \in \mathbb{R}^d$. Therefore, (47) is satisfied because of the "diagonal" structure of f, the second partial derivatives of f_i are non-zero only when $j = k = i$. Therefore,

$$|D^2f(x)yz| \leq \|f''\|_\infty \|W\| |y| |z|$$

by Cauchy-Schwarz inequality. □

The next result concerns the differential of the flow ϕ_t of the vector field N. One has the following general estimates.

Lemma A.2. *Let $\beta \in C^0(\mathbb{R}_+, \mathbb{R}^d)$ and $\gamma \in C^1(\mathbb{R}_+, \mathbb{R})$ be such that $\gamma(0) \geq 0$. Then, for every $t \geq 0$, it holds*

$$\|D\phi_{\gamma(t)}(\beta(t))\| \leq e^{-\gamma(0)\Gamma} e^{\Lambda \int_0^t |\dot\gamma(s)|\, ds}, \quad (48)$$

and

$$\begin{aligned} \|D^2\phi_{\gamma(t)}(\beta(t))\| \leq \Lambda_1 e^{\Lambda \int_0^t |\dot\gamma(\tau)| d\tau} \Big[&\gamma(0) e^{2\Lambda\gamma(0)} \\ &+ \|\dot\gamma\|_\infty t e^{-2\gamma(0)\Gamma} e^{\Lambda \int_0^t |\dot\gamma(\tau)| d\tau} \Big]. \end{aligned} \quad (49)$$

Proof. Let $y \in \mathbb{R}^d$ and $\gamma \in C^1(\mathbb{R}_+, \mathbb{R})$ be such that $\gamma(0) \geq 0$. By differentiating (5) with respect to y, we find

$$D\phi_{\gamma(t)}(y) = D\phi_{\gamma(0)}(y) + \int_0^t \dot\gamma(s) DN(\phi_{\gamma(s)}(y)) D\phi_{\gamma(s)}(y)\, ds.$$

It follows that

$$\begin{aligned} \|D\phi_{\gamma(t)}(y)\| &\leq \|D\phi_{\gamma(0)}(y)\| + \Lambda \int_0^t |\dot\gamma(s)| \|D\phi_{\gamma(s)}(y)\|\, ds \\ &\leq e^{\Lambda \int_0^t |\dot\gamma(s)|\, ds} e^{-\gamma(0)\Gamma} \end{aligned} \quad (50)$$

by (53) and Gronwall's lemma. Let $\beta \in C^0(\mathbb{R}_+, \mathbb{R}^d)$, then for a fixed $t \geq 0$, the map $s \mapsto D\phi_{\gamma(t)}(\beta(s))$ is continuous by composition, and because of (50), we find

$$\|D\phi_{\gamma(t)}(\beta(s))\| \leq e^{\Lambda \int_0^t |\dot\gamma(s)|\, ds} e^{-\gamma(0)\Gamma},$$

which proves (48) by continuity and taking the $\limsup$ as $s \to t$. By differentiating (5) twice with respect to y, we find

$$\begin{aligned} D^2\phi_{\gamma(t)}(y) = &\int_0^t \dot\gamma(s) D^2N(\phi_{\gamma(s)}(y)) D\phi_{\gamma(s)}(y) D\phi_{\gamma(s)}(y)\, ds \\ &+ D^2\phi_{\gamma(0)}(y) + \int_0^t \dot\gamma(s) DN(\phi_{\gamma(s)}(y)) D^2\phi_{\gamma(s)}(y)\, ds, \end{aligned}$$

which yields

$$\begin{aligned} \|D^2\phi_{\gamma(t)}(y)\| \leq &\Lambda_1 e^{-2\gamma(0)\Gamma} e^{2\Lambda \int_0^t |\dot\gamma(\tau)| d\tau} \int_0^t |\dot\gamma(\tau)|\, d\tau \\ &+ e^{\Lambda \int_0^t |\dot\gamma(\tau)| d\tau} \|D^2\phi_{\gamma(0)}(y)\| \end{aligned} \quad (51)$$

by (46), (50) and Gronwall's lemma. Applying (51) with $D^2\phi_t(y)$, i.e., $\gamma(t) = t$, $\gamma(0) = 0$, and $D^2\phi_0(y) = 0$ yields

$$\|D^2\phi_{\gamma(0)}(y)\| \leq \Lambda_1 \gamma(0) e^{2\Lambda\gamma(0)}.$$

It follows that

$$\begin{aligned} \|D^2\phi_{\gamma(t)}(y)\| \leq \Lambda_1 e^{\Lambda \int_0^t |\dot\gamma(\tau)| d\tau} \Big[&\gamma(0) e^{2\Lambda\gamma(0)} \\ &+ \|\dot\gamma\|_\infty t e^{-2\gamma(0)\Gamma} e^{\Lambda \int_0^t |\dot\gamma(\tau)| d\tau} \Big]. \end{aligned} \quad (52)$$

One completes the proof of (49) by arguing as previously. □

It should be noted that when $\gamma(t) = t$, $\|D\phi_t(x)\|$ does not systematically grow with $t \geq 0$. One proves the following by arguing as in (58) below.

Lemma A.3. *For every $t \geq 0$, and all $x \in \mathbb{R}^d$, it holds*

$$\begin{aligned} \|D\phi_t(x)\| &\leq e^{-(\lambda_{\min}(D) - \|W\|)t} \\ \|D\psi_t(x)\| &\geq e^{(\lambda_{\min}(D) - \|W\|)t}. \end{aligned} \quad (53)$$

Appendix B
Proof of some of the results from Section II

A. Proof of Theorem II.2

Proof. Under Assumptions II.1, (4) has a unique solution $x \in C^3([0,T];\mathbb{R}^d)$ by the Cauchy-Lipschitz theory (see, for instance, [6, Chapter 2]). Let us prove that x can be represented by (7). Let $y : t \in [0,T] \mapsto y(t) \in \mathbb{R}^d$ be such that $x(t) = \phi_t(y(t))$. Then, $y(t) = \psi_t(x(t))$, and y is derivable w.r.t. t. Taking the derivative of $x(t) = \phi_t(y(t))$, we find the following by using the chain rule formula, (4), and (5)

$$N(x(t)) + Bu = \dot{x}(t) = N(x(t)) + D\phi_t(y(t))\dot{y}(t).$$

Using (6) and $x(t) = \phi_t(y(t))$, we find $[D\phi_t(y(t))]^{-1} = D\psi_t(x(t))$. It follows that y solves

$$\dot{y}(t) = D\psi_t(x(t))Bu, \qquad y(0) = x^0. \tag{54}$$

Integrating (54) over $[0,t]$ yields (7).

Conversely, let us show that x given by (7) solves (4) and belongs to $C^3([0,T];\mathbb{R}^d)$. First, $x(0) = \phi_0(x^0) = x^0$ and $x \in C^0([0,T];\mathbb{R}^d)$ by composition. Otherwise, there exists $(t_n) \subset [0,T]$, $t_* \in [0,T]$ with $t_n \to t_*$ and $\varepsilon > 0$ such that $|\psi_t(x(t_n)) - \psi_{t_*}(x(t_*))| \geq \varepsilon$. In fact, $x \in C^0([0,T];\mathbb{R}^d)$ if and only if $t \mapsto \psi_t(x(t))$ belongs to $C^0([0,T];\mathbb{R}^d)$ since ψ_t is invertible and C^3 w.r.t. $t \in \mathbb{R}$. We have from (7) that

$$\psi_{t_n}(x(t_n)) - \psi_{t_*}(x(t_*)) = \int_{t_*}^{t_n} D\psi_s(x(s))Bu\,ds$$

which, using (48) with $\gamma(s) = -s$ and $\beta(s) = x(s)$, implies

$$|\psi_{t_n}(x(t_n)) - \psi_{t_*}(x(t_*))| \leq |Bu|e^{\Lambda_1(T-t_*)}|t_n - t_*|.$$

It follows that $|\psi_{t_n}(x(t_n)) - \psi_{t_*}(x(t_*))| \to 0$ as $n \to \infty$, which is inconsistent. Invoking Lebesgue derivation theorem under the integral sign, the r.h.s. of (7) belongs to $C^2([0,T];\mathbb{R}^d)$ by composition and an iterative argument since[1] $\phi_t(\cdot) \in C^2(\mathbb{R}^d)$. Let

$$z(t) = x^0 + \int_0^t D\psi_s(x(s))Bu\,ds$$

so that $x(t) = \phi_t(z(t))$. Deriving (7) w.r.t. t yields

$$\dot{x}(t) = N(\phi_t(z(t))) + Bu = N(x(t)) + Bu \tag{55}$$

by $D\phi_t(z(t))D\psi_t(x(t)) = \mathrm{Id}$. Since the r.h.s. of (55) defines a continuous function in t, one deduces that x given by (7) belongs to $C^3([0,T];\mathbb{R}^d)$ and solves (4). □

Appendix C
Proof of some of the results from Section III-B

We start with the following informative results.

Lemma C.1. *Let $(t,y) \in \mathbb{R}_+ \times \mathbb{R}^d$, $\phi_t(y) = DN(\phi_t(y))$ and $V_t(y) = DN(\psi_t(y))$. Then, it holds*

$$\|e^{tV_t(y)}\| \leq e^{-t(\lambda_{\min}(D) - \|W\|)}. \tag{56}$$

$$\|e^{t\phi_t(y)}\| \leq e^{-t(\lambda_{\min}(D) - \|W\|)}. \tag{57}$$

[1] See, for instance, [17, Chapter 15, Theorem 2].

Proof. First, for a fixed $t \geq 0$ and $(x,y) \in (\mathbb{R}^d)^2$, the map $s \in \mathbb{R}_+ \mapsto e^{tV_s(y)}x \in \mathbb{R}^d$ is continuous by composition. Now fix $s \geq 0$, let $x \in \mathbb{R}^d$, and define for $t \in \mathbb{R}_+$, $g(t) = |e^{tV_s(y)}x|^2$. Then, $g \in C^1(\mathbb{R}_+)$, $g(0) = |x|^2$ and

$$\begin{aligned}\frac{\dot{g}(t)}{2} &= -\left\langle De^{tV_s(y)}x, e^{tV_s(y)}x\right\rangle \\ &\quad + \left\langle WDf(\psi_t(y))e^{tV_s(y)}x, e^{tV_s(y)}x\right\rangle \\ &\leq (-\lambda_{\min}(D) + \|W\|)g(t)\end{aligned} \tag{58}$$

by Cauchy-Schwarz inequality since $\|Df(\psi_t(y))\| \leq 1$. It follows that

$$|e^{tV_s(y)}x|^2 \leq e^{2t(-\lambda_{\min}(D)+\|W\|)}|x|^2.$$

Taking the lim sup as $s \to t$ completes the proof of (56) by continuity. We prove (57) similarly. □

One also has the following result, where the proof follows the same lines as that of Lemma C.1.

Lemma C.2. *Set $A = -D + W$. If $\|W\| \leq \lambda_{\min}(D)$, then*

$$\|e^{tA}\| \leq 1 \qquad \forall t \geq 0.$$

In particular, if $\|W\| < \lambda_{\min}(D)$, then

$$\|e^{tA}\| < 1 \qquad \forall t > 0.$$

In the following lemma, we show that the series in Proposition III.14 are well-defined.

Lemma C.3. *The series (29), (30), and (31) in Proposition III.14 are well-defined in $\mathcal{M}_d(\mathbb{R})$. Moreover, there exists $C = C(t, x^0, D, W, f, B, u) > 0$ such that*

$$\|\zeta_u(t)\| \leq Ce^{3\Lambda t}t^3, \qquad \forall t \geq 0.$$

Proof. Let us prove that

$$\|\xi_u(t)\| \leq \Lambda\Lambda_1|Bu|e^{3\Lambda t}t^3 \tag{59}$$

$$\|\chi_u(t)\| \leq \Lambda_1 \max\left(\Lambda|x^0| + |Bu|, \frac{\|W\|}{\lambda_{\min}(D)}\right)e^{3\Lambda t}t^3 \tag{60}$$

where $\xi_u(t)$ and $\chi_u(t)$ are defined in (30) and (31).

First of all, for every $n \in \mathbb{N}$, one has $1/(n+1) \leq 1$ so that

$$\left\|\sum_{n=0}^{\infty} \frac{t^{n+1}Z_t^n}{(n+1)!}\right\| \leq t\sum_{n=0}^{\infty}\frac{t^n\Lambda^n}{n!} = te^{\Lambda t} \qquad \forall t \geq 0$$

since $\|Z_t^n\| \leq \Lambda^n$ by Lemma A.1. Next, for every $n \geq 1$,

$$\frac{d}{ds}Z_s^n = \sum_{k=1}^{n} Z_s^{k-1}\dot{Z}_sZ_s^{n-k}, \qquad \left\|\frac{d}{ds}Z_s^n\right\| \leq n\Lambda^{n-1}\|\dot{Z}_s\|.$$

Furthermore, $\dot{Z}_s = WD^2f(\psi_s(x(s)))P_sBu$, and, one finds

$$|\xi(t)y| \leq \Lambda_1|Bu|t^3e^{3\Lambda t}|y|$$

by applying (48) with $\gamma(s) = -s$ and $\beta(s) = x(s)$. This completes the proof of (59). Let us prove (60). Firstly, one has $\ddot{x}(s) = DN(x(s))\dot{x}(s)$, $\dot{x}(0) = N(x^0) + Bu$ so that $|\dot{x}(0)| \leq \Lambda|x^0| + |Bu|$ since $N(0) = 0$ and N in Λ-Lipschitz by Lemma A.1. Therefore, by the Cauchy-Schwarz inequality

$$\frac{d}{ds}|\dot{x}(s)| = \frac{\langle\ddot{x}(s), \dot{x}(s)\rangle}{|\dot{x}(s)|} \leq -\lambda_{\min}(D)|\dot{x}(s)| + \|W\|$$

which, by Gronwall's lemma, implies that

$$|\dot{x}(s)| \le \max\left(\Lambda|x^0| + |Bu|, \frac{\|W\|}{\lambda_{\min}(D)}\right), \quad \forall s \ge 0. \quad (61)$$

On the other hand, by applying (49) with $\gamma(s) = -s$ and $\beta(s) = x(s)$, one gets,

$$\|D\psi_s(x(s))\dot{x}(s)\| \le \Lambda_1 s e^{2\Lambda s}|\dot{x}(s)|. \quad (62)$$

One deduces (60) from (31), (61) and (62). □

A. *Proof of Proposition III.14*

Proof. Define for every $n \in \mathbb{N}$, $\eta : t \mapsto \eta(t) = Z_t^n P_t$. Let us show that η is absolutely continuous on $[0,T]$ with value in $\mathcal{M}_d(\mathbb{R})$. First, one has $\eta(\cdot) \in L^1((0,T);\mathcal{M}_d(\mathbb{R}))$. In fact, by applying (48) with $\gamma(t) = -t$ and $\beta(t) = x(t)$, one finds

$$\int_0^T \|\eta(t)\|\,dt \le \Lambda^n \int_0^T e^{\Lambda t}\,dt = \Lambda^{n-1}(e^{\Lambda T} - 1)$$

where $\Lambda = \lambda_{\max}(D) + \|W\|$. Moreover, η is derivable w.r.t. t and $\dot{\eta}(t) = -Z_t P_t + D^2\psi_t(x(t))\dot{x}(t)$ if $n = 0$, while for $n \ge 1$, one has

$$\dot{\eta}(t) = \left(\frac{d}{dt}Z_t^n\right)P_t + Z_t^n \dot{P}_t$$
$$= \sum_{k=1}^n Z_t^{k-1}\dot{Z}_t Z_t^{n-k}P_t - Z_t^n(Z_t P_t - D^2\psi_t(x(t))\dot{x}(t)).$$

By using (61), (62) and applying (48) and (49) with $\gamma(t) = -t$ and $\beta(t) = x(t)$, one finds for $n = 0$,

$$\int_0^T \|\dot{\eta}(t)\|\,dt \le T\Lambda e^{\Lambda T}$$
$$+T^2\Lambda_1 e^{2\Lambda T}\max\left(\Lambda|x^0| + |Bu|, \frac{\|W\|}{\lambda_{\min}(D)}\right).$$

If $n \ge 1$, one uses (46) to obtain $\dot{Z}_t = WD^2 f(\psi_t(x(t)))P_t Bu$ and $\|\dot{Z}_t\| \le \Lambda_1 e^{\Lambda t}|Bu|$. It follows that

$$\int_0^T \|\dot{\eta}(t)\|\,dt \le nT\Lambda^{n-1}\Lambda_1 e^{2\Lambda T}|Bu| + T\Lambda^{n+1}e^{\Lambda T}$$
$$+T^2\Lambda^n\Lambda_1 e^{2\Lambda T}\max\left(\Lambda|x^0| + |Bu|, \frac{\|W\|}{\lambda_{\min}(D)}\right).$$

It follows that $\dot{\eta}(\cdot) \in L^1((0,T);\mathcal{M}_d(\mathbb{R}))$, and that $\eta(\cdot)$ is absolutely continuous on $[0,T]$, which justifies the following integration by parts

$$\psi_t(x(t)) = x^0 + \int_0^t s' P_s Bu\,ds = -\int_0^t \frac{s^2}{2}\left[\frac{d}{ds}Z_s\right]P_s Bu\,ds$$
$$-\int_0^t sD^2\psi_s(x(s))\dot{x}(s)Bu\,ds - \int_0^t \frac{s^2}{2}Z_s D^2\psi_s(x(s))\dot{x}(s)Bu\,ds$$
$$+x^0 + tP_t Bu + \frac{t^2}{2}Z_t P_t Bu + \int_0^t \frac{s^2}{2}Z_s^2 P_s Bu\,ds \quad (63)$$

from which one performs integration by parts on the last integral in (63) and so on to obtain (29), (30), and (31), which are well-defined by Lemma C.3. □

B. *Proof of Proposition III.8*

Proof. Let us introduce

$$v(t) = \int_0^t D\phi_{T-s}(x(s))Bu\,ds, \qquad t \in [0,T]$$

where $x(\cdot)$ is the solution of (4) corresponding to $u \in \mathbb{R}^k$. Applying (48) with $\gamma(t) = T - t$ and $\beta(t) = x(t)$, one finds

$$|v(t)| \le t|Bu|e^{(\Lambda-\Gamma)t}, \qquad t \in [0,T]$$

showing that $v(t) \underset{t\sim 0}{=} \mathcal{O}(t)$. Thus, one deduces from (9) that

$$x(t) \underset{t\sim 0}{=} \phi_t(x^0) + \mathcal{O}(t).$$

It follows that

$$Q_{T-t} := D\phi_{T-t}(x(t)) \underset{t\sim 0}{=} D\phi_{T-t}(\phi_t(x^0)) + \mathcal{O}(t),$$
$$Z_{T-t} := DN(\phi_{T-t}(x(t)) \underset{t\sim 0}{=} A + \mathcal{O}(t),$$
$$D^2\phi_{T-t}(x(t))\dot{x}(t) \underset{t\sim 0}{=} D^2\phi_{T-t}(\phi_t(x^0))N(\phi_t(x^0)) + \mathcal{O}(t),$$

where $A := DN(\phi_T(x^0))$. Using (15) and (16), one finds

$$\kappa_u(T) \underset{T\sim 0}{=} \mathcal{O}(T^4), \qquad \eta_u(T) \underset{T\sim 0}{=} \eta(T) + \mathcal{O}(T^4), \quad (64)$$

$$\eta(T) := \sum_{n=1}^{\infty}\int_0^T \frac{(t-T)^n A^{n-1}}{n!}D^2\phi_{T-t}(\phi_t(x^0))N(\phi_t(x^0))\,dt.$$

Since $DN(\phi_T(x^0))$ is invertible by assumption, it follows from (73) that

$$\eta(T) = \int_0^T \left[e^{(t-T)A} - \mathrm{Id}\right] D\phi_{T-t}(\phi_t(x^0))\,dt -$$
$$\int_0^T \left[e^{(t-T)A} - \mathrm{Id}\right] A^{-1}D\phi_{T-t}(\phi_t(x^0))DN(\phi_t(x^0))\,dt. \quad (65)$$

Set $\quad \Theta_1 := \int_0^T \left[e^{(t-T)A} - \mathrm{Id}\right] D\phi_{T-t}(\phi_t(x^0))\,dt.$

Using integration by parts, one finds

$$\Theta_1 = A^{-1} - e^{-TA}A^{-1}D\phi_T(x^0) - \int_0^T D\phi_{T-t}(\phi_t(x^0))\,dt$$
$$+\int_0^T e^{(t-T)A}A^{-1}D\phi_{T-t}(\phi_t(x^0))DN(\phi_t(x^0))\,dt. \quad (66)$$

since (see, for instance, the proof of Lemma C.4)

$$\frac{d}{dt}D\phi_{T-t}(\phi_t(x^0)) = -D\phi_{T-t}(\phi_t(x^0))DN(\phi_t(x^0)).$$

It follows from (65) and (66) that

$$\eta(T) = \left(\mathrm{Id} - e^{-TA}\right)A^{-1}D\phi_T(x^0) - \int_0^T D\phi_{T-t}(\phi_t(x^0))\,dt. \quad (67)$$

Now, via successive integration by parts, one finds

$$\int_0^T D\phi_{T-t}(\phi_t(x^0))\,dt = \int_0^T t' D\phi_{T-t}(\phi_t(x^0))\,dt$$
$$= T + \frac{T^2}{2}A + \frac{T^3}{6}A^2$$
$$-\int_0^T \frac{t^2}{2}D\phi_{T-t}(\phi_t(x^0))\left[\frac{d}{dt}DN(\phi_t(x^0))\right]\,dt$$
$$+\int_0^T \left(\frac{t^4}{4!}\right)' D\phi_{T-t}(\phi_t(x^0))DN(\phi_t(x^0))^3\,dt \quad (68)$$

and so on to obtain

$$\int_0^T D\phi_{T-t}(\phi_t(x^0))\,dt = \left(e^{TA} - \mathrm{Id}\right) A^{-1} - \underbrace{\sum_{n=1}^{\infty} \int_0^T \frac{t^{n+1} D\phi_{T-t}(\phi_t(x^0))}{(n+1)!} \left[\frac{d}{dt} DN(\phi_t(x^0))^n\right] dt}_{\Lambda_T}. \quad (69)$$

Letting $Z_t := DN(\phi_t(x^0))$, one finds

$$\frac{d}{dt} Z_t^n = \sum_{k=1}^{n} Z_t^{k-1} \dot{Z}_t Z_t^{n-k}, \quad \left\| \frac{d}{dt} Z_t^n \right\| \le n\Lambda^{n-1} \|\dot{Z}_t\|.$$

Furthermore, $\dot{Z}_t = WD^2 f(\phi_t(x^0))N(\phi_t(x^0))$. Therefore,

$$\|\Lambda_T\| \le \Lambda_1 e^{2\Lambda T} |x^0| T^3. \quad (70)$$

Combining (70), (69) and (67), we find

$$\eta(T) \underset{T\sim 0}{=} \left(\mathrm{Id} - e^{-TA}\right) A^{-1} D\phi_T(x^0) - \left(e^{TA} - \mathrm{Id}\right) A^{-1} + \mathcal{O}(T^3). \quad (71)$$

Combining (64) and (71) yields

$$\varphi_u(T) \underset{T\sim 0}{=} \left(\mathrm{Id} - e^{-TA}\right) A^{-1} D\phi_T(x^0) - \left(e^{TA} - \mathrm{Id}\right) A^{-1} + \mathcal{O}(T^3),$$

which complete the proof of (23) since $A := DN(\phi_T(x^0))$. To complete the proof of (24), one uses (18) and (23). □

C. Proof of Theorem III.12

Proof. Let $T \ge \tau$ where $\tau > 0$ be such that the assumption in Theorem III.12 is satisfied, and let $u_\tau \in \mathbb{R}^k$. Then, using (9), the solution $x(\cdot)$ to (4) corresponding to

$$u(t) = \begin{cases} 0 & \text{if} \quad 0 \le t \le T - \tau \\ u_\tau & \text{if} \quad T - \tau < t \le T \end{cases}$$

reads at time $t = T$ as (see, for instance, Remark II.5)

$$x(T) = \phi_T(x^0) + \int_{T-\tau}^{T} D\phi_{T-t}(x(t)) B u_\tau \, dt.$$

Arguing as in the proof of Proposition III.6, one expands

$$x(T) = -\sum_{n=0}^{\infty} \frac{(-\tau)^{n+1} DN(\phi_T(x^0))^n}{(n+1)!} D\phi_\tau(\phi_{T-\tau}(x^0)) B u_\tau + \phi_T(x^0) - \varphi_u(\tau, T) B u_\tau \quad (72)$$

where $\varphi_{u_\tau}(\tau, T) = \kappa_{u_\tau}(\tau, T) + \eta_{u_\tau}(\tau, T)$, with $\kappa_{u_\tau}(\tau, T)$ and $\eta_{u_\tau}(\tau, T)$ defined in (15) and (16), respectively, where the integrals are taken over $[T - \tau, T]$.

If $x(T) = x^1$, one argues as in the proof of Theorem III.7 to obtain that u_τ necessarily solves (28), and conversely. □

The following results were useful.

Lemma C.4. *Let $x \in \mathbb{R}^d$, and let $T > 0$, $t \in [0, T]$. Then,*

$$DN(\phi_T(x))\, D\phi_{T-t}(\phi_t(x)) = D\phi_{T-t}(\phi_t(x))\, DN(\phi_t(x)) + D^2\phi_{T-t}(\phi_t(x))\, N(\phi_t(x)). \quad (73)$$

In particular, when $t = 0$, we obtain

$$DN(\phi_T(x)) = D\phi_T(x)\, DN(x)\, D\phi_T(x)^{-1} + D^2\phi_T(x)\, N(x)\, D\phi_T(x)^{-1}. \quad (74)$$

Proof. Set $y(t) = D\phi_{T-t}(b(t))$ where $b \in C^1(\mathbb{R}_+, \mathbb{R}^d)$. Then, y is derivable by Proposition A.1, and using the chain rule, one finds

$$\dot{y}(t) = -DN(\phi_{T-t}(b(t)))D\phi_{T-t}(b(t)) + D^2\phi_{T-t}(b(t))\dot{b}(t). \quad (75)$$

On the other and, letting $b(t) = \phi_t(x)$ for some $x \in \mathbb{R}^d$, one finds $y(t) = D\phi_{T-t}(\phi_t(x))$ and thus

$$\dot{y}(t) = -D\phi_{T-t}(\phi_t(x))DN(\phi_t(x)). \quad (76)$$

In fact, one finds $D\phi_{T-t}(\phi_t(x))D\phi_t(x) = D\phi_T(x)$ from $\phi_{T-t}(\phi_t(x)) = \phi_T(x)$, so that, after derivation w.r.t., t,

$$\dot{y}(t)D\phi_t(x) = -D\phi_{T-t}(\phi_t(x))DN(\phi_t(x))D\phi_t(x)$$

which yields (76) after right multiplication by $[D\phi_t(x)]^{-1}$. Now, since $\phi_{T-t}(b(t)) = \phi_{T-t}(\phi_t(x)) = \phi_T(x)$ and $\dot{b}(t) = N(\phi_t(x))$, identifying (75) and (76) yields

$$DN(\phi_T(x))D\phi_{T-t}(\phi_t(x)) = D\phi_{T-t}(\phi_t(x))DN(\phi_t(x)) + D^2\phi_{T-t}(\phi_t(x))N(\phi_t(x))$$

completing the proof of (73). Finally, (74) follows by evaluating (73) at $t = 0$, and right multiplication by $[D\phi_T(x)]^{-1}$. □
